# Extensive T cell cross-reactivity between diverse seasonal influenza strains in the ferret model

**DOI:** 10.1038/s41598-018-24394-z

**Published:** 2018-04-17

**Authors:** Adrian J. Reber, Nedzad Music, Jin Hyang Kim, Shane Gansebom, Jufu Chen, Ian York

**Affiliations:** 1 Influenza Division, National Center for Immunization and Respiratory Diseases, Centers for Disease Control and Prevention, 1600, Clifton Road, Atlanta, GA 30329 USA; 2 Present Address: Janssen Research & Development, Spring House, PA 19477 USA; 3 Present Address: Arbutus Biopharma, Warminster, PA 18974 USA

## Abstract

Influenza virus causes widespread, yearly epidemics by accumulating surface protein mutations to escape neutralizing antibodies established from prior exposure. In contrast to antibody epitopes, T cell mediated immunity targets influenza epitopes that are more highly conserved and have potential for cross-protection. The extent of T cell cross-reactivity between a diverse array of contemporary and historical influenza strains was investigated in ferrets challenged with 2009 pandemic H1N1 influenza or the seasonal H3N2 strain, A/Perth/16/2009. Post-challenge cell-mediated immune responses demonstrated extensive cross-reactivity with a wide variety of contemporary and historical influenza A strains as well as influenza B. Responses in peripheral blood were undetectable by 36d post-challenge, but cross-reactivity persisted in spleen. The strongest responses targeted peptides from the NP protein and demonstrated cross-reactivity in both the CD4+ and CD8+ T cell populations. Cross-reactive CD4+ T cells also targeted HA and NA epitopes, while cross-reactive CD8+ T cells targeted internal M1, NS2, and PA. T cell epitopes demonstrated extensive cross-reactivity between diverse influenza strains in outbred animals, with NP implicated as a significant antigenic target demonstrating extensive cross-reactivity for both CD4+ and CD8+ T cells.

## Introduction

Current influenza vaccines are designed to elicit strain-specific neutralizing antibody primarily against hemagglutinin (HA) and neuraminidase (NA), the major surface antigens of influenza viruses. However, antigenic drift within HA of seasonal viruses frequently leads to moderate antigenic mismatch between vaccine and circulating strains^1^. In addition, occasional emergence of viruses with novel HA and NA from animal reservoirs results in pandemic strains with significantly mismatched surface antigens that are resistant to antibody mediated neutralization directed against the seasonal viruses. These issues have led to intense interest in vaccines inducing broadly cross-protective immunity to influenza viruses.

In contrast to antibody epitopes which recognize primarily the hydrophilic, 3-dimensional outer surface of proteins, T cell epitopes are primarily composed of linear 8 to 24 amino acid peptides derived from internal proteins and the internal, hydrophobic regions of external proteins^2,3^. These regions are more conserved between influenza subtypes and could confer immunity to heterologous as well as homologous influenza virus^2–5^.

The human population likely develops T cell responses to influenza proteins relatively early in life^6^ through natural infection or vaccination and are boosted by repeated exposures throughout their lifetime. Current inactivated influenza vaccines are manufactured by exchanging HA and NA proteins from currently circulating influenza A strains with that of the A/Puerto Rico/08/1934 (A/PR/08) “master donor” strain to form the vaccine strains, while influenza B strains utilize the wild-type internal genes. Live-attenuated vaccines use A/Ann Arbor/6/60 and B/Ann Arbor/1/66 (A/Leningrad/134/17/57 and B/USSR/60/69 in some countries) as the master donor strain. Current TIVs are designed primarily to stimulate antibody production, and have been shown to stimulate CD4 T cells as well, a property necessary for effective antibody production. However, due to the inability to actively replicate in cells, these vaccines are less effective at stimulating CD8 T cell responses. Live-attenuated influenza vaccines, on the other hand, are capable of limited replication in cells, more effectively stimulating CD8 as well as CD4 T cells and antibody. T cell mediated responses are therefore based primarily upon cross-reactivity with historical strains in the case of natural infection. T cell mediated protection derived from vaccine exposure relies primarily upon cross-reactivity with the master donor viruses, wild-type B strains (inactivated vaccines), and internal HA and NA epitopes, and are dependent upon the type of vaccine received.

Few studies have evaluated the extent of cell-mediated immune (CMI) cross-reactivity between seasonal influenza strains (see Discussion). Although some studies evaluating T cell cross-reactivity to influenza have been done in the human population, such studies are difficult because of humans’ unknown and complex history of exposure to different influenza subtypes over their lifetime. No laboratory animal model is more extensively utilized across diverse scientific investigations than the mouse model. Mice are convenient from the perspective of animal handling, control over prior exposure, availability of reagents, and control over response variability due to the inbred nature of mouse laboratory strains. However, concern has continued to mount over the last decade as to the broad application of the mouse model to diverse human diseases, compounded by multiple clinical trial failures resulting from studies that had looked promising in mice^7^. The concern over the ability of mice to adequately mimic the diverse array of human diseases, immune responses, and drug toxicity has prompted more effort to develop animal models which more closely reflect the human condition on a disease specific basis^7–9^.

In the case of influenza, mice prove to be a poor model for many types of influenza studies. Although mice can readily be infected with many of the pandemic and avian influenza strains, they are unable to be infected with the majority of human seasonal influenza subtypes, the influenza strains the human population most often contends with, without prior adaptation. Mice further do not reproduce influenza pathogenesis and transmission observed in humans. Indeed, the study presented below would be extremely challenging to perform in mice without first adapting each of the seasonal influenza strains used to the mouse, thus modifying its genetic and protein profile.

The ferret is currently considered the most relevant animal model for influenza infection^10,11^. The ferret can be infected with human influenza strains without prior adaptation and exhibit influenza disease symptoms closely resembling those of humans. Additionally, ferrets are outbred which, although introducing greater variability in disease outcomes and immune responses, more closely resembles the human condition^10,11^. Although the ferret model is not nearly as well characterized as the mouse model, great strides have been made recently to better characterize this model, including the evaluation of reagents for use in this animal model and the development of techniques for the evaluation of ferret immune responses and influenza pathogenesis^12–17^. Here, we evaluated the extent of cross-reactivity of T cell immune responses in outbred ferrets against a diverse array of contemporary and historical seasonal influenza strains.

## Results

### Predicted HLA binding epitopes

To form a basic understanding of influenza T cell epitope cross-reactivity, HLA class I and II binding epitopes derived from viral proteins of a diverse group of influenza viruses were downloaded from the Internet Epitope Database (IEDB: http://www.iedb.org/). Epitopes from the 2009 pandemic H1N1 strain, A/California/08/2009 (A/CA/08), which bound either human HLA-I or HLA-II were first determined, then cross-reactivity with other influenza strains assessed (Fig. S1). The PB1, NP, and M1 proteins appear to represent proteins with the most HLA binding epitopes cross-reactive with A/CA/08. Several HLA binding epitopes spanned the majority of strains selected, while PB1 spanned the entire breadth of diverse influenza strains examined including influenza B strains (Fig. S1). While binding epitope databases provide useful information, these databases tend to be skewed toward the HLA alleles most highly represented within the human population. HLA diversity of the human population is therefore often neglected in these models. To get a better representation of influenza specific T cell cross-reactivity, the ferret model of influenza is useful, providing the control of an experimental setting within an outbred population.

### Cross-reactive T cell responses associated with influenza infection

T cell cross-reactivity associated with influenza infection was assessed *in vivo*, in vaccinated and unvaccinated ferrets. Vaccinated ferrets received a commercially available 2011–2012 trivalent inactivated vaccine (TIV) composed of A/California/07/2009 (a 2009 pandemic H1N1 influenza strain; H1N1pdm09), A/Victoria/210/2009 (A/Perth/16/2009-like), and B/Brisbane/60/2008 (B/Bris/60) influenza virus strains and were subsequently challenged with A/NY/21/2009, a 2009 pandemic H1N1 influenza strain, (TIV/H1N1pdm09). Naïve ferrets were infected with A/NY/21/2009 (Naïve/H1N1pdm09) or the H3N2 strain, A/Perth/16/2009 (A/Pth/16; Naïve/H3N2). Following challenge, ferrets were monitored for changes in body weight and temperature as well as clinical signs of illness. Mock-infected animals did not show clinical signs, while infected ferrets all showed weight loss, fever, and mild clinical signs. Infection was confirmed by recovery of high titers of the expected influenza virus in nasal washes (Fig. S2). Splenocytes were collected from ferrets 14d post-infection and T cell IFN-γ production assessed after stimulation with contemporary and historical influenza strains covering each of the currently circulating subtypes. The virus panel consisted of the contemporary H1N1pdm09, and historical H1N1 strain, A/PR/08. H3N2 strains consisted of the contemporary A/Pth/16 strain and the historical A/Hong Kong/08/1968 (A/HK/08). B strains were composed of the contemporary B/Bris/60 and historical B/Harbin/07/1994 (B/Hbn/07).

**Figure 1:**
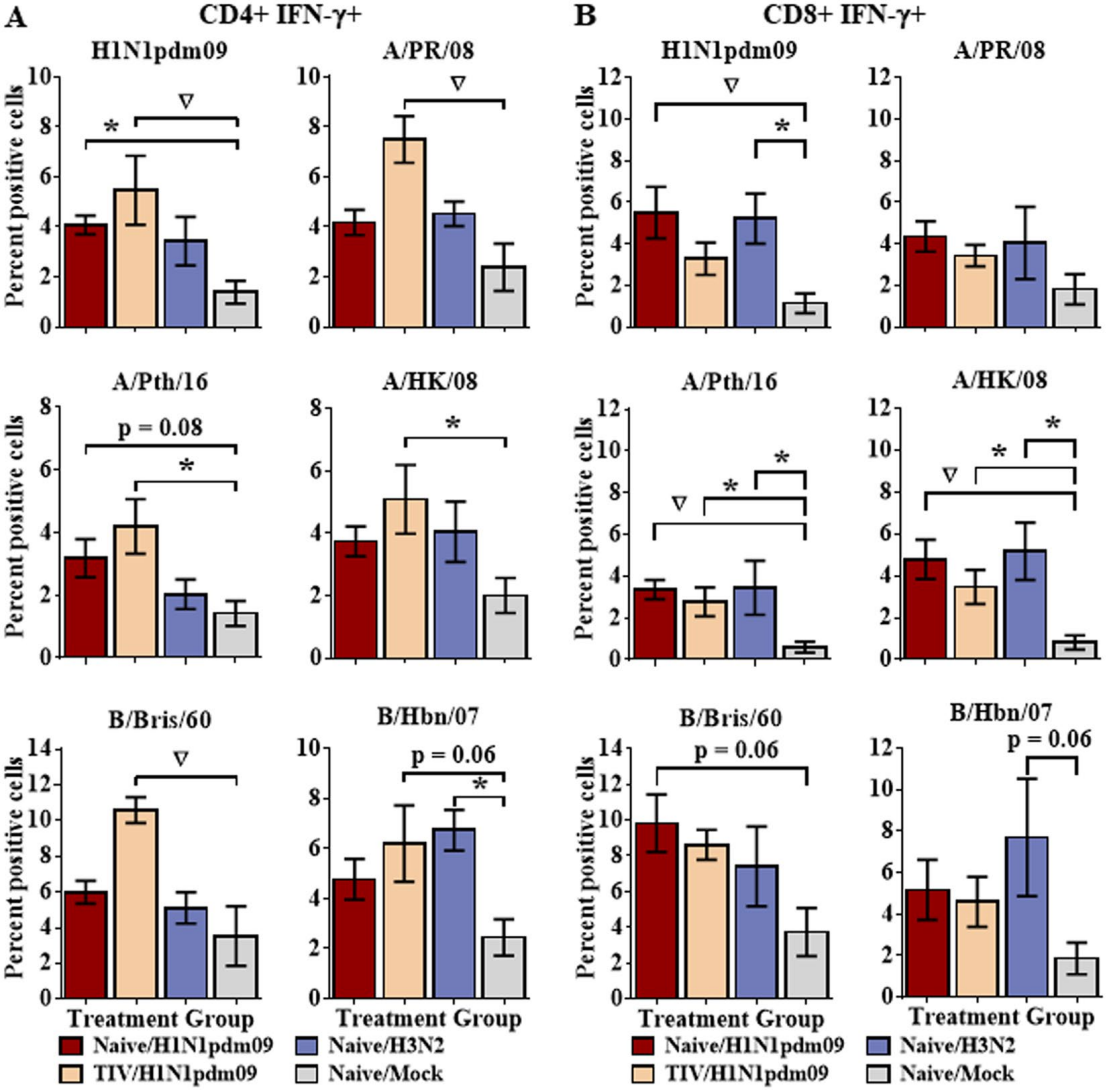
Extensive T cell cross-reactivity between a diverse selection of influenza strains. Naïve ferrets were infected intranasally with H1N1pdm09 strain, A/NY/21/2009 (Naïve/H1N1pdm09, n = 8), the seasonal H3N2 strain, A/Perth/16/2009 (Naïve/H3N2, n = 4), or were mock infected (Naïve/Mock, n = 8). An additional group of ferrets was vaccinated with a commercially available 2011–2012 trivalent inactivated influenza vaccine prior to challenge with A/NY/21/2009 (TIV/H1N1pdm09, n = 8). Splenocytes were collected from ferrets 14d post-infection and stimulated with a diverse array of contemporary and historical influenza strains from each of the currently circulating influenza subtypes. IFN-γ production was assessed by flow cytometry for CD4+ (**A**) and CD8+ (**B**) T cell populations. Significance from the mock infected group is indicated by *p < 0.05, ^∇^p < 0.01. Error bars represent SEM.

Ferrets that had been vaccinated, then challenged with H1N1pdm09 showed easily detectable CD4+ responses to most contemporary and historical strains with which they were stimulated, including H1N1, H3N2, and B strains (Fig. 1A). Unvaccinated H1N1pdm09-infected ferrets also developed CD4 responses to the stimulating 2009 pandemic strain, A/California/08/2009, as well as to the contemporary H3N2 strain, A/Pth/16, although the level of the response tended to be lower than in the vaccinated ferrets. In these ferrets, CD4 responses to other viruses were also detected but did not achieve statistical significance (Fig. 1A). Similarly, CD4 responses in splenocytes from ferrets infected with H3N2 were detectable but did not achieve statistical significance, with the exception of the historical influenza B virus, B/Hbn/07 (Fig. 1A).

CD8 T cell responses also showed extensive cross-reactivity. Unvaccinated ferrets that had been infected with H1N1pdm09 or with H3N2 recognized H1N1pdm09, contemporary and historic H3N2, and influenza B viruses (Fig. 1B). In contrast to the CD4 response, prior vaccination did not appear to enhance the CD8 response.

### Longevity of the cross-reactive T cell response

CD4 and CD8 T cells from splenocytes taken early (14d) after infection showed extensive cross-reactivity between influenza subtypes and even types. Previous studies have suggested that T cell responses may demonstrate broad epitope recognition early in the response, but narrow as the response progresses^18^. To determine the extent of T cell cross-reactivity over time, naïve ferrets were infected with H1N1pdm09 or mock infected, peripheral blood samples taken weekly for 6 weeks, and T cell responses assessed against contemporary and historical viruses.

**Figure 2:**
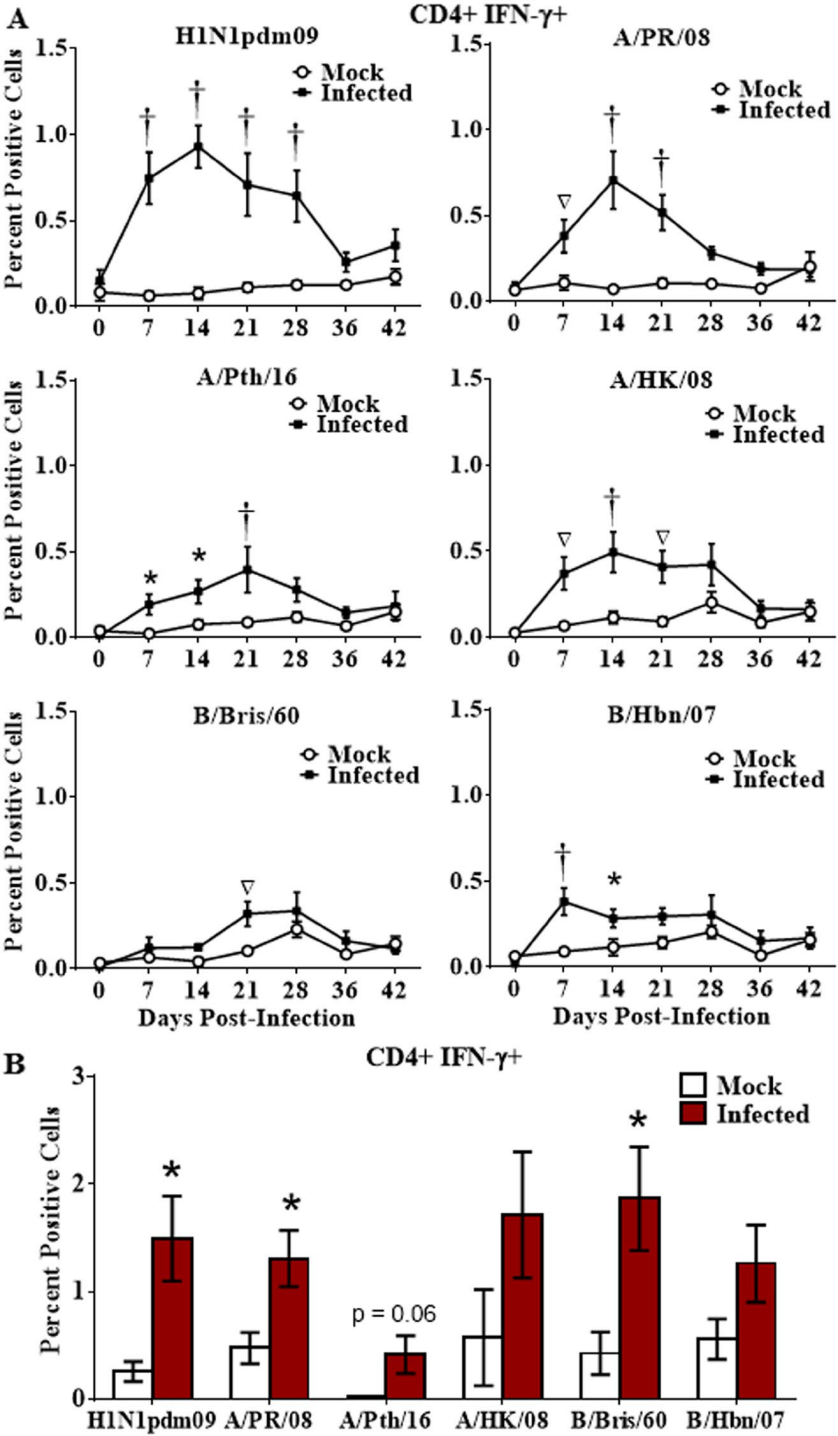
Longevity of the cross-reactive CD4 T cell response. Naïve ferrets were infected intranasally with H1N1pdm09 strain, A/NY/21/2009 (H1N1pdm09, n = 7), or were mock infected (Mock; n = 6). Peripheral blood leukocytes were purified weekly for 6 weeks, and T cell responses assessed by stimulation with a panel of live influenza strains (**A**). Splenocytes were collected 6 weeks post-infection and stimulated with the panel of live influenza strains (**B**). Responding CD4 T cells were assessed for IFN-γ production by flow cytometry. Significance from the mock infected group is indicated by *p < 0.05, ^∇^p < 0.01, ^†^p < 0.001. Error bars represent SEM.

**Figure 3:**
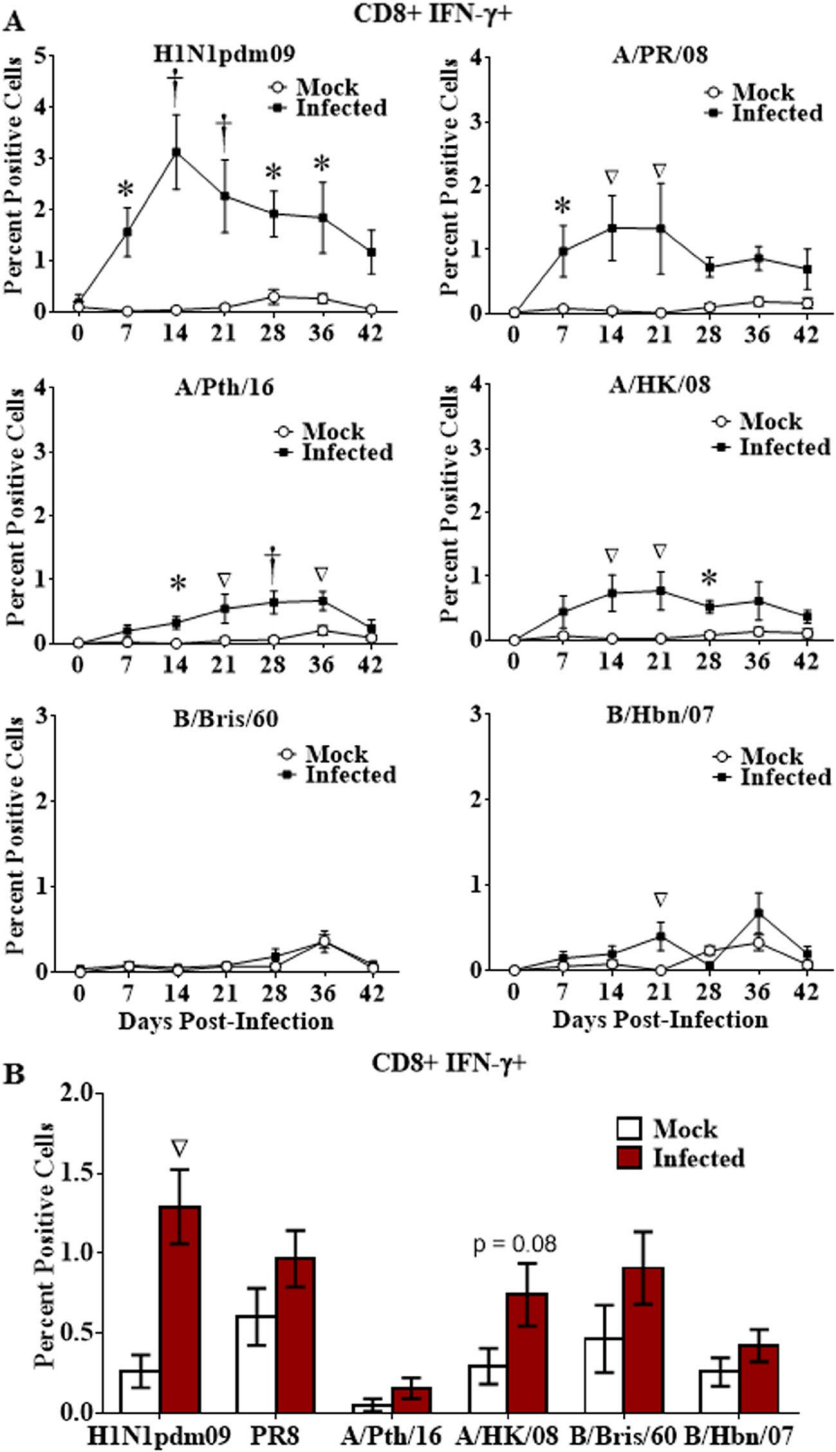
Longevity of the cross-reactive CD8 T cell response. Naïve ferrets were infected intranasally with H1N1pdm09 (H1N1pdm09, n = 7), or mock infected (Mock; n = 6). Peripheral blood leukocytes were purified weekly for 6 weeks, and CD8 T cell responses assessed by stimulation with live virus consisting of a panel of contemporary and historical influenza strains (**A**). Splenocytes were collected 6 weeks post-infection and stimulated with the panel of influenza strains (**B**). Responding CD8 + IFN-γ + T cells were assessed by flow cytometry. Significance from the mock infected group is indicated by *p < 0.05, ^∇^p < 0.01, ^†^p < 0.001. Error bars represent SEM.

The CD4 and CD8 T cell IFN-γ response to H1N1pdm09, the infecting strain, increased in peripheral blood after challenge, peaking 14d post-infection (Figs 2A and 3A), then declined, reaching levels no longer significantly different from mock infected animals by 36d to 42d post-infection (Figs 2A and 3A). Cross-reactivity to all contemporary and historical strains tested (with the exception of one influenza B virus for CD8 T cells, which also poorly recognized the other influenza B virus; Fig. 3A) was observed, but at a lower level than with the challenge strain (Figs 2A and 3A). Cross-reactive responses disappeared from peripheral blood by 28d to 36d post-infection.

While both homologous and cross-reactive T cell responses disappeared from peripheral blood by 36d post-infection, splenocytes collected from these animals at 42d demonstrated that CMI cross-reactivity still persisted in spleen (Figs 2B and 3B). Cross-reactive spleen CD4+ IFN-γ+ T cell responses against the historical H1N1 strain, A/PR/08, and to the contemporary B strain, B/Bris/60, were still apparent at 42d post-infection (Fig. 2B). While cross-reactive CD8+ T cell responses in the spleen were detectable at 42d post-infection, only responses against the infecting strain, H1N1pdm09, were statistically significant (Fig. 3B).

### T cell responses to influenza peptides

Overlapping peptide sequences spanning the entire length of each viral protein were derived from the H1N1pdm09 strain, A/California/07/2009, or the seasonal A/Perth/16/2009-like H3N2 strain, A/Victoria/210/2009. Peptides from each viral strain were combined into peptide pools (p; Fig. S3) for each protein based on the region of the protein from which the peptide was derived. Pools were used to stimulate splenocytes from our initial 4 groups of challenged ferrets (4 from each group) to further define cell-mediated cross-reactive epitopes. Responses significantly greater than the Mock infected group were considered modest responses, while 2-fold greater than the Mock response was considered moderate. Responses significantly greater than 4-fold the Mock responses were considered substantial.

**Figure 4:**
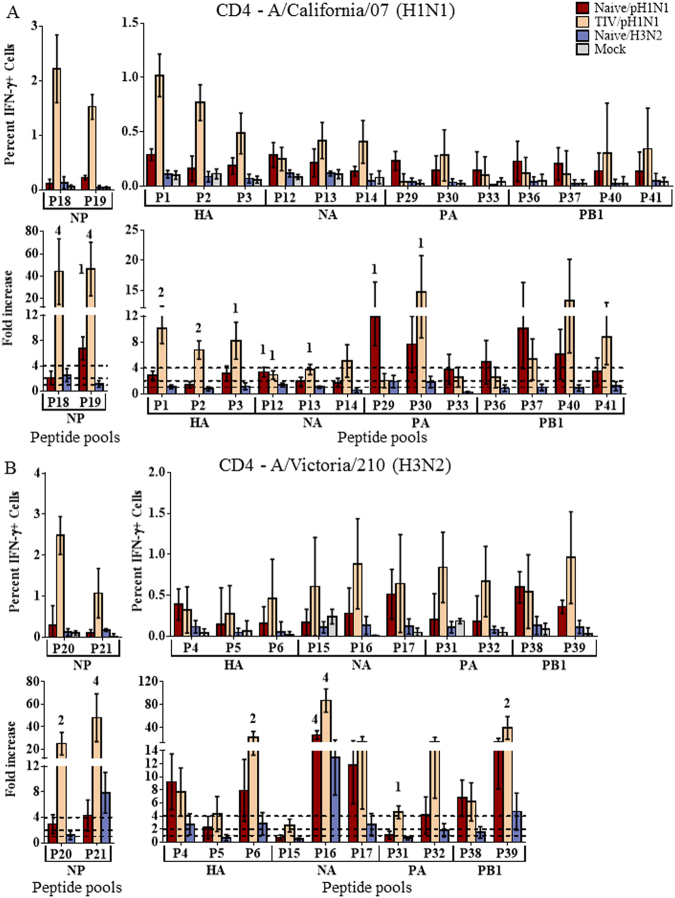
CD4+ T cell responses to influenza peptide pools. Splenocytes were collected from naïve ferrets infected intranasally with A/NY/21/2009 (Naïve/H1N1pdm09, n = 4), A/Perth/16/2009 (Naïve/H3N2, n = 4), or mock infected ferrets (Naïve/Mock, n = 4) 14d post infection. Splenocytes were also collected from ferrets vaccinated with commercial 2011–2012 TIV, challenged with A/NY/21/2009 (TIV/H1N1pdm09, n = 4). Splenocytes were stimulated with peptide pools (p) derived from regions of each influenza protein from A/California/07/2009 (H1N1pdm09; (**A**) or the A/Perth/16/2009-like seasonal H3N2 strain, A/Victoria/210/2009 (**B**). T cells producing IFN-γ in response to peptide stimulation were assessed by flow cytometry. The percent IFN-γ+ cells and fold-increase over mock infected animals are depicted. Responses significantly ^1^greater (p < 0.05) than Mock infected ferrets were considered modest responses, ^2^2-fold greater were considered moderate, and ^4^4-fold greater were considered substantial. Dotted lines indicate 1-, 2-, and 4-fold increases over mock infected controls. Error bars represent SEM.

**Figure 5:**
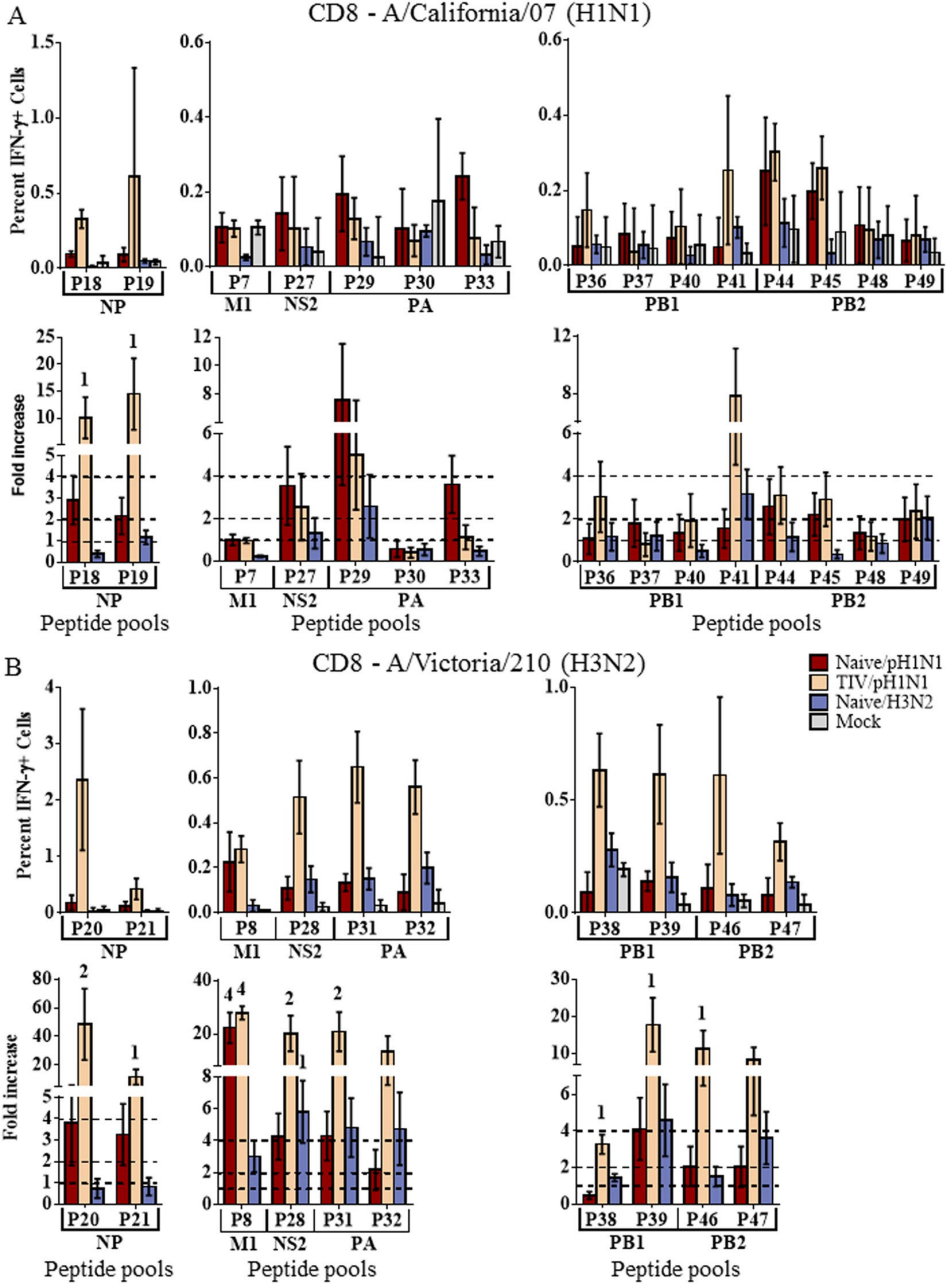
CD8+ T cell responses to influenza peptide pools. Splenocytes were collected from naïve ferrets infected intranasally with A/NY/21/2009 (Naïve/H1N1pdm09, n = 4), A/Perth/16/2009 (Naïve/H3N2, n = 4), or mock infected ferrets (Naïve/Mock, n = 4) 14d post infection. Splenocytes were also collected from ferrets vaccinated with commercial 2011–2012 TIV, challenged with A/NY/21/2009 (TIV/H1N1pdm09, n = 4). Splenocytes were stimulated with peptide pools (p) derived from regions of each influenza protein from A/California/07/2009 (H1N1pdm09; (**A**) or the A/Perth/16/2009-like seasonal H3N2 strain, A/Victoria/210/2009 (**B**). T cells producing IFN-γ in response to peptide stimulation were assessed by flow cytometry. The percent IFN-γ + cells and fold-increase over mock infected animals are depicted. Responses significantly ^1^greater (p < 0.05) than Mock infected ferrets were considered modest responses, ^2^2-fold greater were considered moderate, and ^4^4-fold greater were considered substantial. Dotted lines indicate 1-, 2-, and 4-fold increases over mock infected controls. Error bars represent SEM.

The most significant peptide responses were generally associated with the NP protein. Vaccinated ferrets challenged with H1N1pdm09 (TIV/H1N1pdm09) demonstrated substantial CD4+ responses to pools composing both the N-terminal and C-terminal halves of the H1N1pdm09 NP protein (Fig. 4A). Modest CD8+ responses were also demonstrated to the H1N1pdm09 NP protein (Fig. 5A). Although challenged with H1N1pdm09, these ferrets also demonstrated significant cross-reactive responses to NP of the H3N2 strain, A/Victoria/210/2009 (Figs 4B and 5B). Substantial cross-reactive CD4+ responses were demonstrated to the C-terminal portion of A/Victoria/210/2009 NP, and moderate responses to the N-terminal portion. Moderate cross-reactive CD8+ responses to the N-terminal portion of the A/Victoria/210/2009 NP protein were also apparent, with modest responses to the C-terminal portion (Fig. 5B). Although not significant (p < 0.07), heightened CD4+ responses were observed to homologous NP epitopes shared by both H1N1pdm09 and A/Victoria/210/2009 (Fig. S4A).

In addition to the NP protein, other protein epitopes drove significant cross-reactive CD4+ responses. Individual ferret responses to each peptide pool are represented in the Supplemental data (Figs S5 and S6). Epitopes from the NA protein drove substantial cross-reactive CD4+ responses to A/Victoria/210/2009 in ferrets challenged with H1N1pdm09, both vaccinated and unvaccinated (Fig. 4B). Moderate cross-reactive responses were observed to epitopes from the C-terminal portions of HA and PB1 (Fig. 4B). Although heightened CD8+ T cell responses were observed to the A/Victoria/210/2009 NA protein, in vaccinated ferrets challenged with H1N1pdm09, the majority of these responses did not reach significance (Fig. S4B). However, substantial CD8+ cross-reactivity was observed to the A/Victoria/210/2009 M1 protein (Fig. 5B). Moderate CD8+ cross-reactive responses were also observed to epitopes from the NS2 and PA proteins (Fig. 5B).

**Figure 6:**
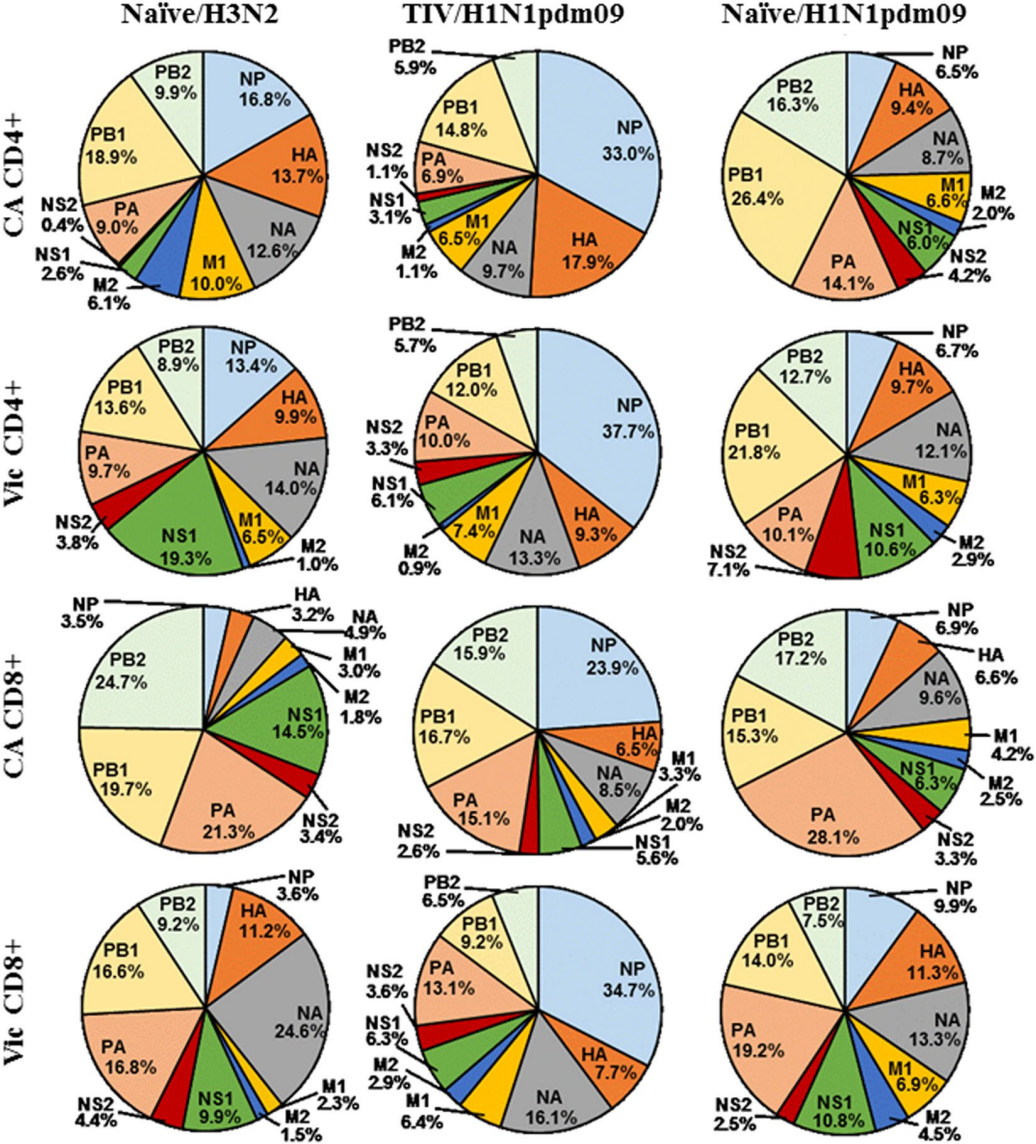
Characterization of influenza specific T cell target proteins. T cell responses targeting each of the influenza proteins based on the results of our peptide stimulation were expressed as the proportion of the total T cell response.

Peptide responses were assessed to determine the proportion of the total T cell response devoted to each of the viral proteins (Fig. 6). Unvaccinated ferrets challenged with either H1N1pdm09 or A/Pth/16 were generally dominated by PB1, PB2 and PA responses whether they were to homologous or heterologous proteins. Vaccination had a substantial effect on T cell responses. Not only did vaccinated ferrets have considerably more responding T cells, but also shifted toward NP dominated T cell responses (Fig. 6) recognizing homologous and heterologous NP protein.

Homologies of viral proteins which tended to dominate the influenza T cell responses were analyzed for influenza strains both contemporary and historical, constructing p-distance heat maps to demonstrate conservation of these proteins. HA and NA proteins demonstrated high homology based primarily on influenza subtype (Fig. S7). The internal proteins NP and PB1 demonstrated more conserved homology with influenza strains with the smallest p-distance clustering within influenza type A or type B, regardless of being contemporary or historical strains (Fig. S7). Of all the proteins examined, PB1 demonstrated the best homology between influenza A and B types.

## Discussion

Influenza causes widespread epidemics, impacting the world’s population on a yearly basis. Current influenza vaccines confer moderate levels of protection against circulating strains by inducing neutralizing antibodies. However, as influenza viruses accumulate point mutations in the receptor binding portion of HA, the virus’s major surface protein and primary neutralizing target^1^, antibodies once capable of protecting against influenza infection are rendered ineffective against strains in subsequent seasons. This antigenic drift, as well as the threat of pandemics from novel influenza subtypes of zoonotic origin, has led to interest in developing vaccines which inducing more broadly cross-reactive immunity conferring protection against a wider range of influenza strains and subtypes. One class of potential broadly cross-reactive vaccine is based on T cell mediated immunity, which tends to target more conserved epitopes than do antibodies.

T cell responses to influenza viruses are less well understood than are the antibody responses, but there is some evidence for broadly cross-reactive T cell immunity. Unfortunately, much of the research that has been performed has concentrated on the pandemic and avian strains of influenza, which although they pose significant threats are rare events in the human population. This has left a substantial gap in the understanding of T cell cross-reactivity between seasonal influenza strains, strains the human population contends with on an annual basis. Previous studies have demonstrated the existence of T cells in humans capable of recognizing H1N1pdm09 viruses prior to its circulation in humans in 2009, presumably established by exposure to cross-reactive seasonal influenza strains^19–23^. Similarly, studies have demonstrated human CD4+ and CD8+ T cell immunity to avian H5N1 and H7N9 influenza viruses, strains to which the majority of the population have not been exposed, but that are considered to pose a significant pandemic threat^24–26^. Similar studies in mice have confirmed CD4+ and CD8+ T cell cross-reactivity with pandemic and “pre-pandemic” influenza strains, and further established their ability to confer protection^27–29^. Studies in ferrets, considered the most relevant animal model for influenza, demonstrated that ferrets infected with a single exposure to a number of seasonal influenza A viruses was capable of inducing varying degrees of protection to H1N1pdm09, with multiple exposures resulting in even better protection^30^.

Adults are typically infected with multiple different strains and subtypes of influenza virus throughout their lifetime, leading to a complex immune response that potentially includes both newly-recruited and memory cells. Ferrets are outbred animals whose response to influenza virus infection is generally very similar to that of humans, and they are therefore considered the best model for human influenza infections; however, reagents that allow measurement of ferret cell-mediated immunity are just becoming available^10,11,16,31^. We observed extensive cross-reactivity in infected ferrets between seasonal influenza A subtypes, and even between antigenically distant influenza A and B strains. Prior vaccination enhanced the CD4+ T cell response (Fig. 1A). This was probably mainly through a prime-boost effect, but the response may also become broader due to initial priming with homologous epitopes from the vaccine master donor virus, B strain epitopes, and broader range of HAs and NAs in the trivalent vaccine. In contrast to the CD4 response, vaccinated animals challenged with H1N1pdm09 tended to have lower CD8+ responses compared to unvaccinated animals. Pre-formed, neutralizing antibodies induced by prior vaccination prevent infection by live virus *in vivo*, shifting antigen presentation toward the exogenous antigen presenting pathway and thereby favoring CD4 responses while dampening CD8 responses (Fig. 1A,B).

Human peripheral blood T cell responses may wax and wane during the first few weeks after influenza infection with the repertoire of T cell specificities changing over time^18^. This was also reflected in ferrets with T cell responses to most influenza strains (both homologous and heterologous) peaking at 14d post-challenge and declining to undetectable levels over the next several weeks (Figs 2A and 3A). However, extensive cross-reactivity observed still exists in the spleen after responses in the peripheral blood have declined to undetectable levels (Figs 2B and 3B).

Our previous work examining trafficking of leukocytes in ferrets following influenza virus challenge demonstrated differential trafficking kinetics of CD4 and CD8 T cells to peripheral blood, spleen, and a variety of lymphoid and non-lymphoid tissues^16^. Considering the functional purpose of the spleen as a primary lymphoid tissue in comparison to the peripheral blood which primarily serves for conveyance from one part of the body to the other, it is probably not unanticipated that influenza responsive T cells persisted in the spleen (including cross-reactive T cells) after declining in the peripheral blood, once infecting virus had been cleared. Considering the extensive homing of T cells previously observed to draining lymph nodes^16^, it is likely that cross-reactive T cells also persisted in those regions of the body. Recent advances in immunology have allowed the further subdivision of T cells into naïve, central memory, and effector memory compartments which reflect functional and homing properties of the cells. It is likely that the differences observed are also reflected in differences in these T cell subsets, however, their associated markers are not yet available for the ferrets as they are for mice and humans.

Characterization of T cell epitopes using peptide pools showed strong T cell responses were stimulated to influenza NP in vaccinated animals (Figs 4A and 5A). NP epitopes have previously been shown to be dominant epitopes for T cell responses in humans^18,25,32^, and the fact that NP also appears to be immunodominant in outbred ferrets suggests this may be due to characteristics of NP itself rather than particular MHC binding preferences. Interestingly, in our experiments, vaccination was shown to not only enhance the T cell response, but also modulate the response such that homologous and heterologous NP specific responses dominated (Fig. 6).

NP proteins are highly conserved across influenza subtypes (Fig. S7). Ferret CD4 and CD8 T cells from ferrets that were vaccinated and infected with H1N1pdm09 showed strong cross-reactivity with the NP protein of A/Victoria/210/2009 (H3N2; Figs 4B and 5B). These responses were presumably initiated by the NP proteins in the inactivated vaccine, derived from the A/PR/08 master donor strain and/or the wild type influenza B strain, B/Brisbane/60/2008, with cross-reactive memory cells being boosted by the H1N1pdm09 wild type NP protein during infection. Cross-reactivity with A/Victoria/210/2009 was likely influenced by cross-reactivity with one or all of these influenza strains. The immunodominant nature of NP and extensive conservation may make the NP protein a good candidate for stimulating cross-reactive CD4 and CD8 T cell responses to diverse influenza strains.

Influenza causes widespread epidemics on a yearly basis due to its ability to accumulate point mutations in its HA protein, the primary neutralizing target, thus escaping previously established neutralizing antibodies. For this reason, the HA protein is often pictured as a highly variable, continuously evolving immunological target. In fact, HA point mutations accumulate primarily in regions under selective pressure from neutralizing antibodies, while the remainder of the protein remains relatively well conserved^1^, allowing recognition by T cells. Vaccinated H1N1pdm09-infected ferrets had CD4+ T cells that cross-reacted well with HA from A/Victoria/210/2009 (Fig. 4B). Substantial cross-reactivity was also observed to the A/Victoria/210/2009 NA protein in both vaccinated and unvaccinated animals challenged with H1N1pdm09 (Fig. 4B). It has recently been speculated that CD4+ T cells targeting HA epitopes may be particularly important for the production of neutralizing antibodies^21,33^.

CD8+ T cell responses are not as easily assessed using peptide stimulation due to the inefficiency of cross-presenting exogenous peptides into the endogenous antigen presentation pathway. Despite the lower and more variable CD8+ T cell responses observed with peptide stimulation, significant responses were observed. In contrast to the CD4+ T cell responses, strong CD8+ T cell responses were not observed to the HA protein (Fig. S4B). Some strong cross-reactive CD8+T cell responses were observed to A/Victoria/210/2009 NA protein, but were highly variable between animals. More consistent cross-reactive CD8+ T cell responses were observed to the internal proteins, with substantial cross-reactivity to the A/Victoria/210/2009 M1 protein and moderate NS2 and PA responses (Fig. 5B). These responses contrast to the CD4+ cross-reactive epitopes which occurred primarily in the surface HA and NA proteins and highlight the differences between CD4+ and CD8+ epitope recognition. Only the NP protein showed strong, cross-reactive responses for both CD4+ and CD8+ T cells.

Extensive T cell cross-reactivity between influenza strains considered to be serologically diverse were demonstrated in the outbred ferret model. These results highlight the differences between CD4+ and CD8+ T cell cross-reactivity. Only the NP protein demonstrated epitopes cross-reactive within both the CD4+ and CD8+ T cell populations. T cell responses to NP epitopes were further shown to be modulated by influenza vaccination. In the search for broader influenza protection, T cell mediated immunity presents itself as a viable candidate with the influenza NP protein as a potential primary target.

## Methods

### Viruses

Viruses were propagated in the allantoic cavity of 10 day-old fertile embryonated chicken eggs (Hy-line, Mansfield, GA) at 34 °C for 48 h (72 h for B viruses). Virus containing allantoic fluid was harvested and frozen at −80 °C until use. Stocks were titered by plaque assay using Madin-Darby Canine Kidney cells and expressed as plaque forming units (pfu).

### Predicted Cross-reactive T Cell Epitopes and Protein Homology

HLA class I and II binding epitopes specific for viral proteins of multiple influenza strains were downloaded from the Internet Epitope Database in November 2016 (IEDB: http://www.iedb.org/), and duplicate sequences were removed. Proteins expressed by the indicated viruses were downloaded from GISAID (http://platform.gisaid.org/) and aligned using MUSCLE^34^. HLA class I and II epitopes were then compared to protein sequences, and the start and end positions of perfect matches (ignoring gaps introduced during alignment) were identified.

For homology assessment for NP, PB1, HA, and NA, genes were aligned with MUSCLE and p-distances were calculated using MEGA7^35^.

### Animals, Vaccination, and Challenge

The study was conducted in accordance with the Animal Welfare Act regulations of the United States Department of Agriculture and Public Health Service Policy on Humane Care and Use of Laboratory Animals administered by the National Institutes of Health. Animal experiments were performed under a Centers for Disease Control and Prevention Institutional Animal Care and Use Committee approved protocol (#2611YORFERC) and conducted in an Association for Assessment and Accreditation of Laboratory Animal Care International accredited animal facility. Animal welfare was monitored daily, and animal handling performed under light anesthesia (ketamine/xylazine); all efforts were made to minimize animal stress and suffering.

Male Fitch ferrets approximately 6 months of age (Triple F Farms, Sayre, PA), randomized by weight, were used throughout the project. All animals were confirmed serologically negative for currently circulating human influenza H1, H3 and type B viruses prior to initiation of the study.

To assess T cell cross-reactivity associated with influenza viral infection *in vivo*, ferrets were divided into four groups. One group was vaccinated intramuscularly with an adult human dose (0.5 ml or 15 µg of HA) per ferret of commercially available 2011–2012 TIV composed of A/California/07/2009, A/Victoria/210/2009 (A/Perth /16/2009-like), and B/Brisbane/60/2008 influenza virus strains (Fluarix; GlaxoSmithKline Biologicals, Research Triangle Park, NC), and boosted 21d later. Standard vaccine HA concentrations were confirmed for each vaccine strain using a standard HA single radial immunodiffusion assay^36^ (not shown; reference serums and antigens obtained from the Center for Biologics Evaluation and Research of the U.S. Food and Drug Administration, Kensington, MD). These vaccinated ferrets were infected intranasally 14d post-boost with 1 × 10^6^ pfu of live A/NY/21/2009, the 2009 pandemic H1N1 strain (TIV/H1N1pdm09, n = 8). Naïve ferrets were mock vaccinated with phosphate-buffered saline (PBS) and infected with H1N1pdm09 (Naïve/H1N1pdm09, n = 8); the seasonal H3N2 strain, A/Pth/16 (Naïve/H3N2, n = 4) or were mock infected (Naïve/Mock, n = 8) with sterile egg allantoic fluid diluted in PBS to be equivalent to the concentration of allantoic fluid in the virus challenge. Infection was confirmed by exhibition of symptoms of disease including weight loss (not shown), and shedding of high titers of influenza virus in nasal secretions. Animals were euthanized 14d post-infection by intracardiac administration of an overdose of euthanasia agent containing pentobarbital. Spleens were collected, minced for purification of splenocytes and frozen in liquid nitrogen for later assessment of T cell responses.

To assess longevity of T cell cross-reactivity, naïve ferrets were challenged intranasally with 1 × 10^6^ pfu of A/NY/21/2009 (H1N1pdm09; n = 7) or mock infected with 0.5 ml of sterile egg allantoic fluid, diluted in PBS (Mock; n = 6). Peripheral blood samples were collected weekly, peripheral blood leukocytes purified, and T cell responses assessed by stimulation with a panel of live influenza strains. Animals were euthanized 42d post-infection and splenocytes collected as outlined above.

### Peptide libraries

Peptides (New England Peptide, Gardner, MA) were derived from the proteome of the H1N1pdm09 strain, A/California/07/2009, or the seasonal A/Perth/16/2009-like H3N2 strain, A/Victoria/210/2009. Peptide sequences were composed of 18 amino acids with a 10 amino acid overlap with each subsequent peptide, spanning the entire length of each viral protein. Individual peptides from each viral strain were combined into peptide pools for each protein based on the region of the protein from which the peptide was derived. Homologous peptide sequences between the two strains were combined into their own peptide pools for each viral protein (Fig. S3). The majority of the peptide pools consisted of 22–24 peptides with two pools as small as 9 peptides. The entire viral proteomes of the two viruses composed a total of 50 peptide pools. A pool of irrelevant peptides derived from CD74 antigen, egg white lysozyme, Beta-Amyloid, and coagulation factor was used as a stimulation control (New England Peptide).

### Assessment of T cell Responses

Cells were stimulated with a panel of live viruses consisting of contemporary and historical strains from each of the currently circulating subtypes or with peptide pools. Our live virus panel consisted of the contemporary H1N1pdm09, and historical H1N1 strain, A/PR/08. H3N2 strains consisted of the contemporary A/Pth/16 strain and the historical A/HK/08. B strains were composed of the contemporary B/Bris/60 and historical B/Hbn/07.

Cells were stimulated overnight with 1 multiplicity of infection of live-virus or peptide such that the final concentration of each peptide within the pool was ∼5 µg/ml. Brefeldin A (Golgi Plug; BD Biosciences, San Diego, CA) was added to cultures for the last 6hrs to block Golgi transport. Cells were stained with a live/dead stain (Life Technologies, Grand Island, NY), then stained with monoclonal antibodies recognizing CD4 (60003-MM02-P, clone: 02, Sino Biological, Beijing, China), CD8 (48-0086-42, clone: OKT-8, eBioscience, San Diego, CA), and IFN-γ (MCA1783A647, clone: CC302, AbD Serotec, Raleigh, NC)^31^, and analyzed using a Canto II Flow Cytometer (BD Biosciences).

### Statistical analysis

Initial responses to live virus stimulation were evaluated by Kruskal-Wallis multiple comparison test comparing each group against mock infected controls using Graphpad Prism (Graphpad Software, Inc., La Jolla, CA). Time-course analyses for longevity responses were assessed using a linear mixed model with repeated measures. T cell responses to peptide pool stimulation were determined by first log_10_ transforming percent positive cells, then estimating the differences in means using repeated measures linear mixed models^37–39^. Back-transformation of differences between the treatment groups and mock infected group yielded response ratios (fold increase). These analyses were performed using SAS software (SAS Institute Inc., Cary, NC).

### Data availability statement

The datasets associated with the current study are available from the corresponding authors upon reasonable request.

### Disclaimer

The findings and conclusions in this report are those of the authors and do not necessarily represent the views of the Centers for Disease Control and Prevention or the Agency for Toxic Substances and Disease Registry.

## Electronic supplementary material

Supplementary Figures
