# Supplementary material for: Extensive T cell cross-reactivity between diverse seasonal influenza strains in the ferret model
Source: Sci Rep. 2018 Apr 17;8:6112. doi: 10.1038/s41598-018-24394-z (PMC5904180; doi:10.1038/s41598-018-24394-z)
Supplement: Supplementary file 1 — Supplementary Figures [file 41598_2018_24394_MOESM1_ESM.pdf]

Extensive T cell cross-reactivity between diverse seasonal influenza strains in the ferret model

Adrian J Reber, Nedzad Music, Jin Hyang Kim, Shane Gansebom, Jufu Chen, Ian York

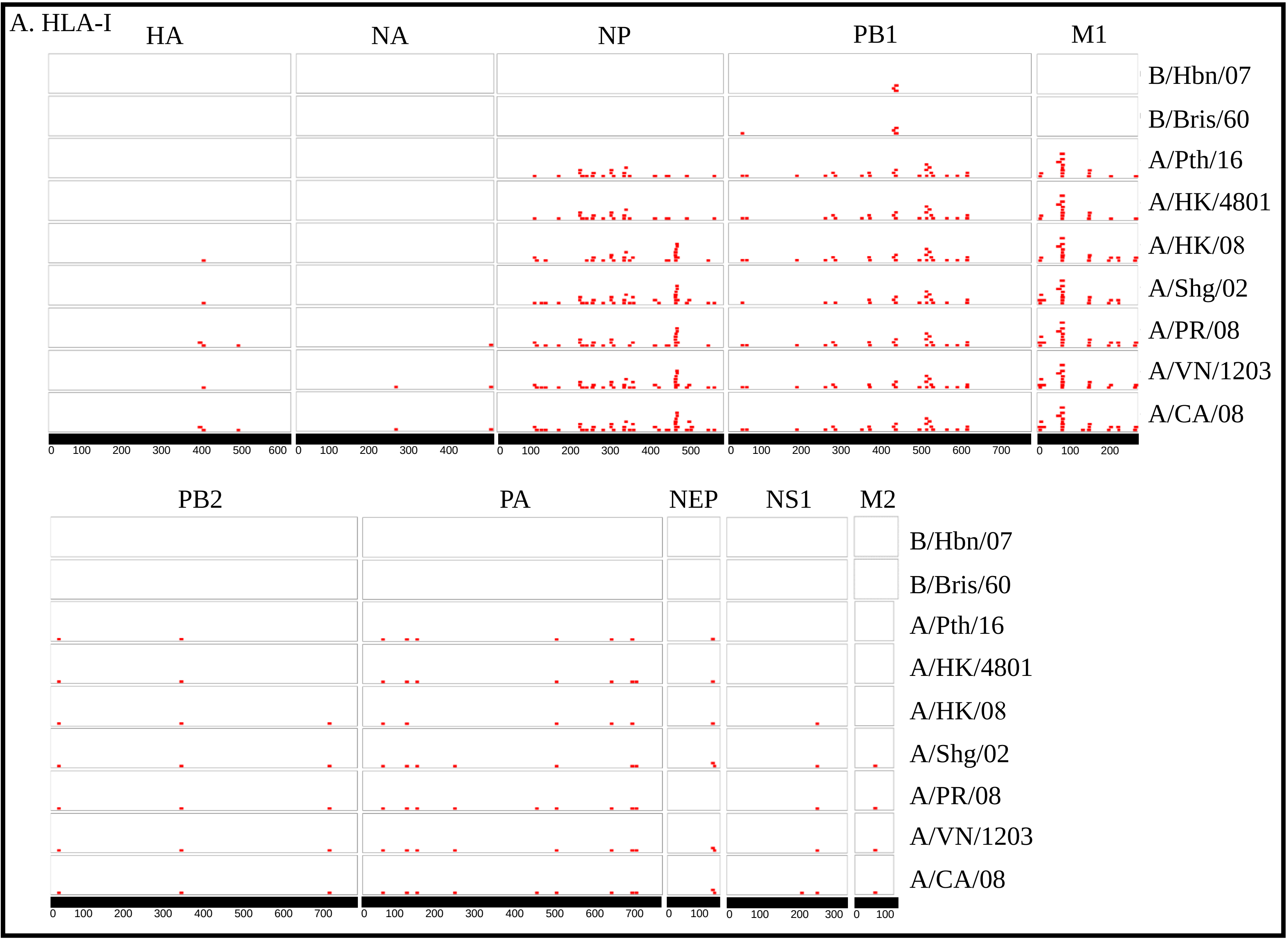
A. HLA-I
HA
NA
NP
PB1
M1
B/Hbn/07
B/Bris/60
A/Pth/16
A/HK/4801
A/HK/08
A/Shg/02
A/PR/08
A/VN/1203
A/CA/08
PB2
PA
NEP
NS1
M2

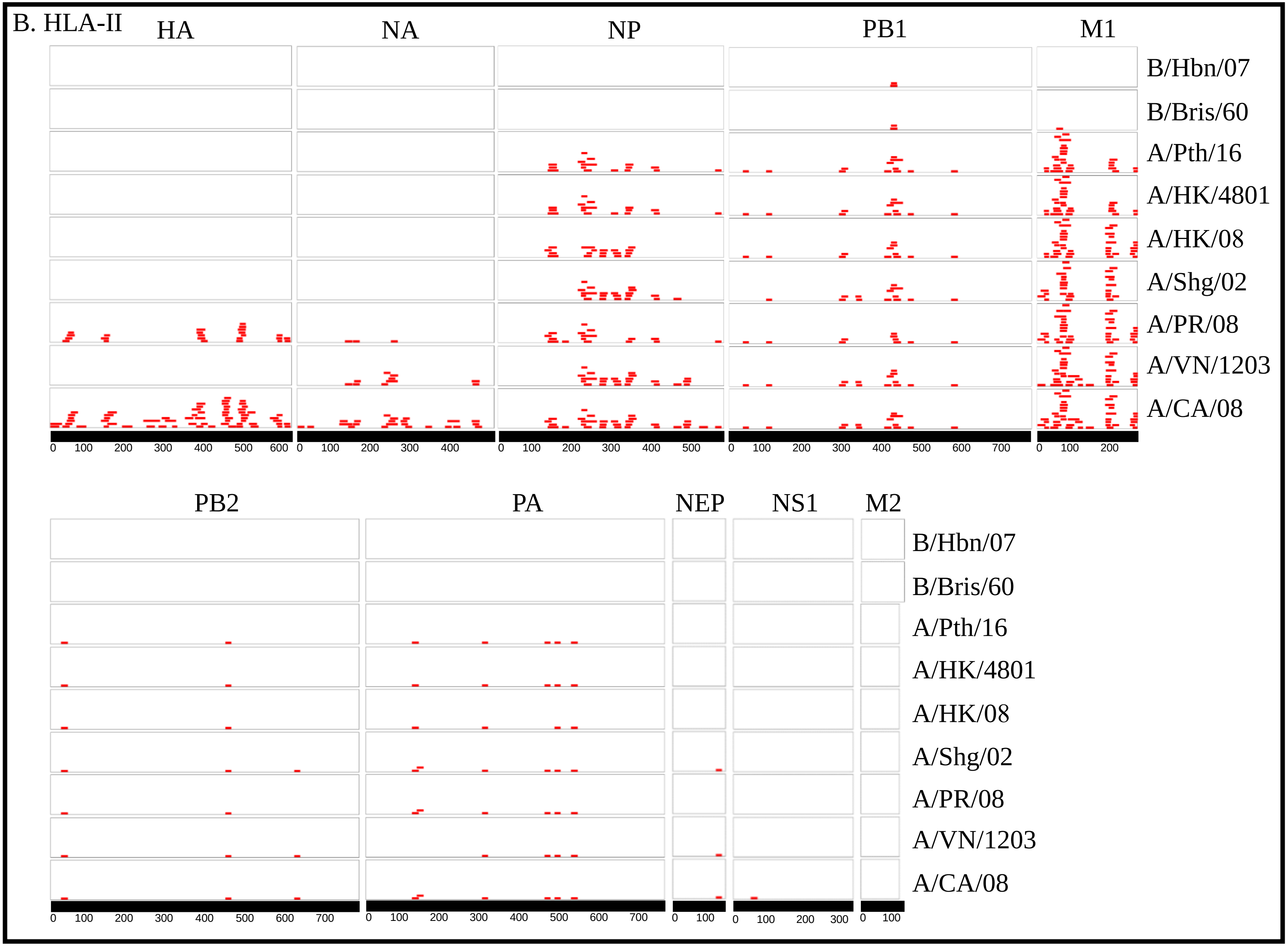

B. HLA-II
HA
NA
NP
PB1
M1
B/Hbn/07
B/Bris/60
A/Pth/16
A/HK/4801
A/HK/08
A/Shg/02
A/PR/08
A/VN/1203
A/CA/08
0 100 200 300 400 500 600
0 100 200 300 400
0 100 200 300 400 500
0 100 200 300 400 500 600 700
0 100 200
PB2
PA
NEP
NS1
M2
B/Hbn/07
B/Bris/60
A/Pth/16
A/HK/4801
A/HK/08
A/Shg/02
A/PR/08
A/VN/1203
A/CA/08
0 100 200 300 400 500 600 700
0 100 200 300 400 500 600 700
0 100
0 100 200 300
0 100

Figure. S1. A/California/08/2009 Human Leukocyte Antigen class I and II binding epitopes cross-reactive with a diverse selection of influenza strains. Known HLA class I (A) and HLA class II (B) binding epitopes derived from a diverse group of influenza viruses spanning both human and animal strains were downloaded from the Internet Epitope Database (IEDB: http://www.iedb.org/). The epitopes from the 2009 pandemic H1N1 strain, A/California/08/2009 (A/CA/08), which bound either human HLA-I or HLA-II were first determined, then cross-reactivity of those epitopes with other influenza strains assessed. For a broader comparison of potential cross-reactivity, in addition to influenza strains used within the study, comparisons to the avian influenza strains A/Shanghai/02/2013 (A/Shg/02; H7N9) and A/Vietnam/1203/2004 (A/VN/1203; H5N1) were performed as well as to the currently circulating human H3N2 strain A/Hong Kong/4801/2014 (A/HK/4801). HLA binding epitopes from each protein are depicted in red. Epitopes from the entire influenza proteome are depicted. The y-axis represents the amino acid sequence designation.

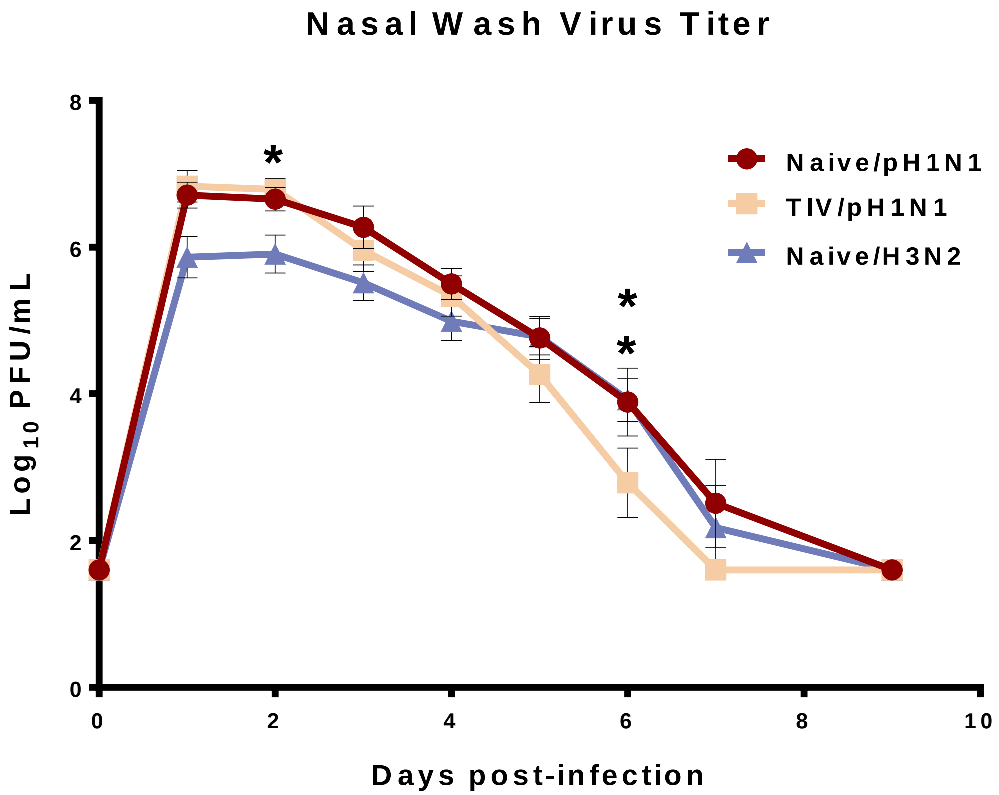

Figure S2. Nasal Wash Titer. Two weeks after booster vaccination, ferrets were challenged intranasally with 10^6 PFU of H1N1pdm09 (TIV/pH1N1). Unvaccinated ferrets were challenged intranasally with 10^6 PFU of H1N1pdm09 (Naive/pH1N1) or with the seasonal H3N2 strain, A/Perth/16/2009 (Naïve/H3N2). Nasal washes were obtained on days 0–9 and virus titers in the nasal washes were measured. Error bars standard error of the mean (SEM). Significance is indicated by * $p < 0.05$.

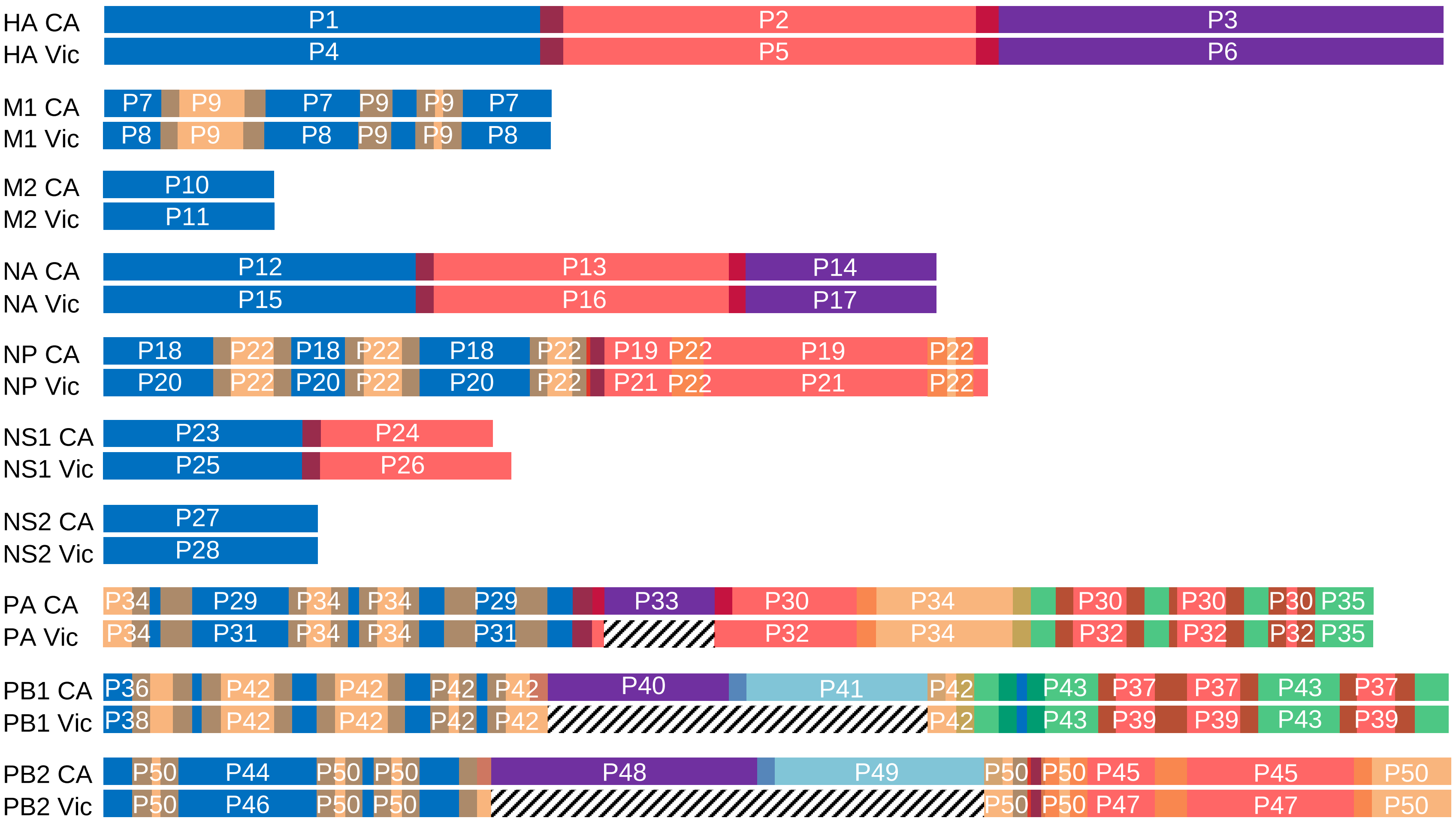
HA CA
HA Vic
M1 CA
M1 Vic
M2 CA
M2 Vic
NA CA
NA Vic
NP CA
NP Vic
NS1 CA
NS1 Vic
NS2 CA
NS2 Vic
PA CA
PA Vic
PB1 CA
PB1 Vic
PB2 CA
PB2 Vic
P1 P2 P3
P4 P5 P6
P7 P9 P9 P7 P9 P9 P7
P8 P9 P8 P9 P9 P8
P10
P11
P12 P13 P14
P15 P16 P17
P18 P22 P18 P22 P18 P22 P19 P22 P19 P22
P20 P22 P20 P22 P20 P22 P21 P22 P21 P22
P23 P24
P25 P26
P27
P28
P34 P29 P34 P34 P29 P33 P30 P34 P30 P30 P30 P35
P34 P31 P34 P34 P31 P32 P34 P32 P32 P32 P35
P36 P42 P42 P42 P42 P40 P41 P42 P43 P37 P37 P43 P37
P38 P42 P42 P42 P42 P42 P43 P39 P39 P43 P39
P50 P44 P50 P50 P48 P49 P50 P50 P45 P45 P50
P50 P46 P50 P50 P50 P50 P47 P47 P50

Strain	Protein	Sequence	Peptide Pool
CA07	HA	MKAILVVLLYTFATANAD	P1
CA07	HA	LYTFATANADTLCIGYHA	P1
CA07	HA	ADTLCIGYHANNSTDTVD	P1
CA07	HA	HANNSTDTVDTVLEKNVT	P1
CA07	HA	VDTVLEKNVTVTHSVNLL	P1
CA07	HA	VTVTHSVNLLEDKHNGKL	P1
CA07	HA	LLEDKHNGKLCKLRGVAPL	P1
CA07	HA	KLCKLRGVAPLHLGKCNI	P1
CA07	HA	APLHLGKCNIAGWILGNPE	P1
CA07	HA	NIAGWILGNPECESLSTAS	P1
CA07	HA	NPECESLSTASSWSYIVE	P1
CA07	HA	TASSWSYIVETPSSDNGT	P1
CA07	HA	VETPSSDNGTCYPGDFID	P1
CA07	HA	GTCYPGDFIDYEELREQL	P1
CA07	HA	IDYEELREQLSSVSSFER	P1
CA07	HA	QLSSVSSFERFEIFPKTS	P1
CA07	HA	ERFEIFPKTSSWPNHDSN	P1
CA07	HA	TSSWPNHDSNKGVTAACPH	P1
CA07	HA	SNKGVTAACPHAGAKSFY	P1
CA07	HA	CPHAGAKSFYKNLIWLVK	P1
CA07	HA	FYKNLIWLVKKGNSYPKLS	P1
CA07	HA	VKKGNSYPKLSKSYINDK	P1
CA07	HA	KLSKSYINDKGKEVLVLW	P1
CA07	HA	DKGKEVLVLWGIHHPSTS	P2
CA07	HA	LWGIHHPSTSADQQSLYQ	P2
CA07	HA	TSADQQSLYQNADAYVFV	P2
CA07	HA	YQNADAYVFVGSSRYSKK	P2
CA07	HA	FVGSSRYSKKFKPEIAIR	P2
CA07	HA	KKFKPEIAIRPKVRDQEG	P2
CA07	HA	IRPKVRDQEGRMNYYWTL	P2
CA07	HA	EGRMNYYWTLVEPGDKIT	P2
CA07	HA	TLVEPGDKITFEATGNLV	P2
CA07	HA	ITFEATGNLVVPRYAFAM	P2
CA07	HA	LVVPRYAFAMERNAGSGI	P2
CA07	HA	AMERNAGSGIIISDTPVH	P2
CA07	HA	GIIISDTPVHDCNTTCQT	P2
CA07	HA	VHDCNTTCQTPKGAINTS	P2
CA07	HA	QTPKGAINTSLPFQNIHPI	P2
CA07	HA	TSLPFQNIHPITIGKCPKY	P2
CA07	HA	HPITIGKCPKYVKSTKLR	P2
CA07	HA	PKYVKSTKLRLATGLRNI	P2
CA07	HA	LRLATGLRNIPSIQSRGL	P2
CA07	HA	NIPSIQSRGLFGAIAGFI	P2
CA07	HA	GLFGAIAGFIEGGWTGMV	P2
CA07	HA	FIEGGWTGMVDGWYGYHH	P2
CA07	HA	MVDGWYGYHHQNEQGSGY	P2
CA07	HA	HHQNEQGSGYAADLKSTQ	P3
CA07	HA	GYAADLKSTQNAIDEITN	P3
CA07	HA	TQNAIDEITNKVNSVIEK	P3
CA07	HA	TNKVNSVIEKMNTQFTAV	P3
CA07	HA	EKMNTQFTAVGKEFNHLE	P3
CA07	HA	AVGKEFNHLEKRIENLNK	P3
CA07	HA	LEKRIENLNKKVDDGFLD	P3

CA07	HA	NKKVDDGFLDIWTYNAEL	P3
CA07	HA	LDIWTYNAELLVLLENER	P3
CA07	HA	ELLVLLENERTLDYHDSN	P3
CA07	HA	ERTLDYHDSNVKNLYEKV	P3
CA07	HA	SNVKNLYEKVRSQLKNNA	P3
CA07	HA	KVRSQLKNNAKEIGNGCF	P3
CA07	HA	NAKEIGNGCFEFYHKCDN	P3
CA07	HA	CFEFYHKCDNTCMESVKN	P3
CA07	HA	DNTCMESVKNGTYDYPKY	P3
CA07	HA	KNGTYDYPKYSEEAKLNR	P3
CA07	HA	KYSEEAKLNREEIDGVKL	P3
CA07	HA	NREEIDGVKLESTRIYQI	P3
CA07	HA	KLESTRIYQILAIYSTVA	P3
CA07	HA	QILAIYSTVASSLVLVVS	P3
CA07	HA	VASSLVLVVSLGAISFWM	P3
CA07	HA	VSLGAISFWMCSNGSLQC	P3
CA07	HA	AISFWMCSNGSLQCRICI	P3
VIC210	HA	MKTIIALSYILCLVFAQK	P4
VIC210	HA	YILCLVFAQKLPGNDNST	P4
VIC210	HA	QKLPGNDNSTATLCLGHH	P4
VIC210	HA	STATLCLGHHAVPNGTIV	P4
VIC210	HA	HHAVPNGTIVKTITNDQI	P4
VIC210	HA	IVKTITNDQIEVTNATEL	P4
VIC210	HA	QIEVTNATELVQNSSTGEI	P4
VIC210	HA	ELVQNSSTGEICDSPHQI	P4
VIC210	HA	GEICDSPHQILDGKNCTLI	P4
VIC210	HA	QILDGKNCTLIDALLGDPQ	P4
VIC210	HA	TLIDALLGDPQCDGFQNK	P4
VIC210	HA	DPQCDGFQNKKWDLFVER	P4
VIC210	HA	NKKWDLFVERSKAYSNCY	P4
VIC210	HA	ERSKAYSNCYPYDVPDYA	P4
VIC210	HA	CYPYDVPDYASLRSLVAS	P4
VIC210	HA	YASLRSLVASSGTLEFNN	P4
VIC210	HA	ASSGTLEFNNESFNWTGV	P4
VIC210	HA	NNESFNWTGVTQNGTSSAC	P4
VIC210	HA	GVTQNGTSSACIRRSKNS	P4
VIC210	HA	SACIRRSKNSFFSRLNWL	P4
VIC210	HA	NSFFSRLNWLTNLNFKYPA	P4
VIC210	HA	WLTNLNFKYPALNVTMPN	P4
VIC210	HA	YPALNVTMPNNEQFDKLY	P4
VIC210	HA	PNNEQFDKLYIWGVHHPV	P5
VIC210	HA	LYIWGVHHPVTDKDQIFL	P5
VIC210	HA	PVTDKDQIFLYAQASGRI	P5
VIC210	HA	FLYAQASGRITVSTKRSQ	P5
VIC210	HA	RITVSTKRSQQTVIPNIG	P5
VIC210	HA	SQQTVIPNIGSRPRVRNI	P5
VIC210	HA	IGSRPRVRNIPSRISIYW	P5
VIC210	HA	NIPSRISIYWTIVKPGDI	P5
VIC210	HA	YWTIVKPGDILLINSTGN	P5
VIC210	HA	DILLINSTGNLIAPRGYF	P5
VIC210	HA	GNLIAPRGYFKMQSGKSS	P5
VIC210	HA	YFKMQSGKSSIMRSDAPI	P5
VIC210	HA	SSIMRSDAPIGKCNSECI	P5
Perth16	HA	PIGKCNSECITPNGSIPN	P5

Perth16	HA	CITPNGSIPNDKPFQNVNR	P5
Perth16	HA	PNDKPFQNVNRITYGACPR	P5
Perth16	HA	VNRITYGACPRYVKQNTL	P5
Perth16	HA	CPRYVKQNTLKLATGMRN	P5
Perth16	HA	TLKLATGMRNVPEKQTRG	P5
VIC210	HA	NVPEKQTRGIFGAIAGFI	P5
VIC210	HA	GIFGAIAGFIENGWEGMV	P5
VIC210	HA	FIENGWEGMVDGWYGFRH	P5
VIC210	HA	MVDGWYGFRHQNSEGRGQ	P5
VIC210	HA	RHQNSEGRGQAADLKSTQ	P6
VIC210	HA	GQAADLKSTQAAIDQING	P6
VIC210	HA	TQAAIDQINGKLNRLIGK	P6
VIC210	HA	NGKLNRLIGKTNEKFHQI	P6
VIC210	HA	GKTNEKFHQIEKEFSEVE	P6
VIC210	HA	QIEKEFSEVEGRIQDLEK	P6
VIC210	HA	VEGRIQDLEKYVEDTKID	P6
VIC210	HA	EKYVEDTKIDLWSYNAEL	P6
VIC210	HA	IDLWSYNAELLVALENQH	P6
VIC210	HA	ELLVALENQHTIDLTDSE	P6
VIC210	HA	QHTIDLTDSEMNKLFEKT	P6
VIC210	HA	SEMNKLFEKTKKQLRENA	P6
VIC210	HA	KTKKQLRENAEDMGNGCF	P6
VIC210	HA	NAEDMGNGCFKIYHKCDN	P6
VIC210	HA	CFKIYHKCDNACIGSIRN	P6
VIC210	HA	DNACIGSIRNGTYDHNVY	P6
VIC210	HA	RNGTYDHNVYRDEALNNR	P6
VIC210	HA	VYRDEALNNRFQIKGVEL	P6
VIC210	HA	NRFQIKGVELKSGYKDWI	P6
VIC210	HA	ELKSGYKDWILWISFAIS	P6
VIC210	HA	WILWISFAISCFLLCVAL	P6
VIC210	HA	ISCFLLCVALLGFIMWAC	P6
VIC210	HA	ALLGFIMWACQKGNIRCN	P6
VIC210	HA	GFIMWACQKGNIRCNICI	P6
CA07	M1	MSLLTEVETYVLSIIPSG	P7
CA07	M1	TYVLSIIPSGPLKAEIAQ	P7
CA07	M1	SGPLKAEIAQRLESVFAG	P7
CA07	M1	AQRLESVFAGKNTDLEAL	P7
CA07	M1	QNALNGNGDPNNMDRAVK	P7
CA07	M1	DPNNMDRAVKLYKKLKRE	P7
CA07	M1	VKLYKKLKREITFHGAKE	P7
CA07	M1	REITFHGAKEVSLSYSTG	P7
CA07	M1	KEVSLSYSTGALASCMGL	P7
CA07	M1	TGALASCMGLIYNRMGTV	P7
CA07	M1	GLIYNRMGTVTTEAAFGL	P7
CA07	M1	TVTTEAAFGLVCATCEQI	P7
CA07	M1	QIADSQHRSHRQMATTTN	P7
CA07	M1	SHRQMATTTNPLIRHENR	P7
CA07	M1	TNPLIRHENRMVLASTTA	P7
CA07	M1	AGSSEQAAEAMEVANQTR	P7
CA07	M1	EAMEVANQTRQMVHAMRT	P7
CA07	M1	TRQMVHAMRTIGTHPSSS	P7
CA07	M1	RTIGTHPSSSAGLKDDLL	P7
CA07	M1	SSAGLKDDLLENLQAYQK	P7
CA07	M1	LLENLQAYQKRMGVQMQR	P7

CA07	M1	ENLQAYQKRMGVQMQRFK	P7
VIC210	M1	MSLLTEVETYVLSIVPSG	P8
VIC210	M1	TYVLSIVPSGPLKAEIAQ	P8
VIC210	M1	SGPLKAEIAQRLEDVFAG	P8
VIC210	M1	AQRLEDVFAGKNTDLEAL	P8
VIC210	M1	QNALNGNGDPNNMDKAVK	P8
VIC210	M1	DPNNMDKAVKLYRKLKRE	P8
VIC210	M1	VKLYRKLKREITFHGAKE	P8
VIC210	M1	REITFHGAKEIALSYSAG	P8
VIC210	M1	KEIALSYSAGALASCMGL	P8
VIC210	M1	AGALASCMGLIYNRMGAV	P8
VIC210	M1	GLIYNRMGAVTTEVAFGL	P8
VIC210	M1	AVTTEVAFGLVCATCEQI	P8
VIC210	M1	QIADSQHRSHRQMVATTN	P8
VIC210	M1	SHRQMVATTNPLIKHENR	P8
VIC210	M1	TNPLIKHENRMVLASTTA	P8
VIC210	M1	AGSSEQAAEAMEIASQAR	P8
VIC210	M1	EAMEIASQARQMVQAMRA	P8
VIC210	M1	ARQMVQAMRAIGTHPSSS	P8
VIC210	M1	RAIGTHPSSSTGLRDDLL	P8
VIC210	M1	SSTGLRDDLLENLQTYQK	P8
VIC210	M1	LLENLQTYQKRMGVQMQR	P8
VIC210	M1	ENLQTYQKRMGVQMQRFK	P8
CA07/VIC210	M1	AGKNTDLEALMEWLKTRPI	P9
CA07/VIC210	M1	ALMEWLKTRPILSPLTKG	P9
CA07/VIC210	M1	RPILSPLTKGILGFVFTL	P9
CA07/VIC210	M1	KGILGFVFTLTVPSERGL	P9
CA07/VIC210	M1	TLTVPSERGLQRRRFVQN	P9
CA07/VIC210	M1	GLQRRRFVQNALNGNGDPN	P9
CA07/VIC210	M1	GLVCATCEQIADSQHRSH	P9
CA07/VIC210	M1	NRMVLASTTAKAMEQMAG	P9
CA07/VIC210	M1	TAKAMEQMAGSSEQAAEA	P9
CA07	M2	MSLLTEVETPTRSEWECR	P10
CA07	M2	TPTRSEWECRCSDSSDPL	P10
CA07	M2	CRCSDSSDPLVIAANIIG	P10
CA07	M2	PLVIAANIIGILHLILWI	P10
CA07	M2	IGILHLILWITDRLFFKC	P10
CA07	M2	WITDRLFFKCIYRRFKYG	P10
CA07	M2	KCIYRRFKYGLKRGPSTE	P10
CA07	M2	YGLKRGPSTEGVPESMRE	P10
CA07	M2	TEGVPESMREEYQQEQQS	P10
CA07	M2	REEYQQEQQSAVDVDDGH	P10
CA07	M2	QQSAVDVDDGHFVNIELE	P10
VIC210	M2	MSLLTEVETPIRNEWGCR	P11
VIC210	M2	TPIRNEWGCRCNDSSDPL	P11
VIC210	M2	CRCNDSSDPLVVAANIIG	P11
VIC210	M2	PLVVAANIIGILHLILWI	P11
VIC210	M2	IGILHLILWILDRLFFKC	P11
VIC210	M2	WILDRLFFKCVYRLFKHG	P11
VIC210	M2	KCVYRLFKHGLKRGPSTE	P11
VIC210	M2	HGLKRGPSTEGVPESMRE	P11
VIC210	M2	TEGVPESMREEYRKEQQN	P11
VIC210	M2	REEYRKEQQNAVDADDSH	P11
VIC210	M2	QQNAVDADDSHFVSIELE	P11

CA07	NA	MNPNQKIITIGSVCMTIG	P12
CA07	NA	TIGSVCMTIGMANLILQI	P12
CA07	NA	IGMANLILQIGNIISIWI	P12
CA07	NA	QIGNIISIWISHSIQLGN	P12
CA07	NA	WISHSIQLGNQNQIETCN	P12
CA07	NA	GNQNQIETCNQSVITYEN	P12
CA07	NA	CNQSVITYENNTWVNQTY	P12
CA07	NA	ENNTWVNQTYVNISNTNF	P12
CA07	NA	TYVNISNTNFAAGQSVVS	P12
CA07	NA	NFAAGQSVVSVKLAGNSSL	P12
CA07	NA	VSVKLAGNSSLCPVSGWA	P12
CA07	NA	SSLCPVSGWAIYSKDNSV	P12
CA07	NA	WAIYSKDNSVRIGSKGDV	P12
CA07	NA	SVRIGSKGDVFVIREPFI	P12
CA07	NA	DVFVIREPFISCSPLECR	P12
CA07	NA	FISCSPLECRTFFLTQGA	P12
CA07	NA	CRTFFLTQGALLNDKHSN	P12
CA07	NA	GALLNDKHSNGTIKDRSPY	P12
CA07	NA	SNGTIKDRSPYRTLMSCPI	P12
CA07	NA	SPYRTLMSCPIGEVPSPY	P12
CA07	NA	CPIGEVPSPYNSRFESVA	P12
CA07	NA	PYNSRFESVAWSASACHD	P12
CA07	NA	VAWSASACHDGINWLTIG	P13
CA07	NA	HDGINWLTIGISGPDNGA	P13
CA07	NA	IGISGPDNGAVAVLKYNG	P13
CA07	NA	GAVAVLKYNGIITDTIKS	P13
CA07	NA	NGIITDTIKSWRNNILRT	P13
CA07	NA	KSWRNNILRTQESECACV	P13
CA07	NA	RTQESECACVNGSCFTVM	P13
CA07	NA	CVNGSCFTVMTDGPSNGQ	P13
CA07	NA	VMTDGPSNGQASYKIFRI	P13
CA07	NA	GQASYKIFRIEKGKIVKS	P13
CA07	NA	RIEKGKIVKSVEMNAPNY	P13
CA07	NA	KSVEMNAPNYHYEECSCY	P13
CA07	NA	NYHYEECSCYPDSSEITC	P13
CA07	NA	CYPDSSEITCVCRDNWHG	P13
CA07	NA	TCVCRDNWHGSNRPWVSF	P13
CA07	NA	HGSNRPWVSFNQNLEYQI	P13
CA07	NA	SFNQNLEYQIGYICSGIF	P13
CA07	NA	QIGYICSGIFGDNPRPND	P13
CA07	NA	IFGDNPRPNDKTGSCGPV	P13
CA07	NA	NDKTGSCGPVSSNGANGV	P13
CA07	NA	PVSSNGANGVKGFSYKYG	P13
CA07	NA	GVKGFSYKYGNGVWIGRT	P13
CA07	NA	YGNGVWIGRTKSISSRNG	P14
CA07	NA	RTKSISSRNGFEMIWDPN	P14
CA07	NA	NGFEMIWDPNGWTGTDNNF	P14
CA07	NA	PNGWTGTDNNFSIKQDIV	P14
CA07	NA	NNFSIKQDIVGINEWSGY	P14
CA07	NA	IVGINEWSGYSGSFVQHPE	P14
CA07	NA	GYSGSFVQHPELTGLDCI	P14
CA07	NA	HPELTGLDCIRPCFWVEL	P14
CA07	NA	CIRPCFWVELIRGRPKEN	P14
CA07	NA	ELIRGRPKENTIWTSGSS	P14

CA07	NA	ENTIWTSGSSISFCGVNS	P14
CA07	NA	SSISFCGVNSDTVGWSWPD	P14
CA07	NA	NSDTVGWSWPDGAELPFT	P14
CA07	NA	TVGWSWPDGAELPFTIDK	P14
VIC210	NA	MNPNQKIITIGSVSLTIS	P15
VIC210	NA	TIGSVSLTISTICFFMQI	P15
VIC210	NA	ISTICFFMQIAILITTVT	P15
VIC210	NA	QIAILITTVTLHFKQYEF	P15
VIC210	NA	VTLHFKQYEFNSPPNNQV	P15
VIC210	NA	EFNSPPNNQVMLCEPTII	P15
VIC210	NA	QVMLCEPTIIERNITEIV	P15
VIC210	NA	IIERNITEIVYLTNTTIE	P15
VIC210	NA	IVYLTNTTIEKEICPKLA	P15
VIC210	NA	IEKEICPKLAEYRNWSKPQ	P15
VIC210	NA	LAEYRNWSKPQCDITGFA	P15
VIC210	NA	KPQCDITGFAPFSKDNSI	P15
VIC210	NA	FAPFSKDNSIRLSAGGDI	P15
VIC210	NA	SIRLSAGGDIWVTREPYV	P15
VIC210	NA	DIWVTREPYVSCDPDKCY	P15
VIC210	NA	YVSCDPDKCYQFALGQGT	P15
VIC210	NA	CYQFALGQGTTLNNVHSN	P15
VIC210	NA	GTTLNNVHSNNTVRDRTPY	P15
VIC210	NA	SNNTVRDRTPYRTLLMNEL	P15
VIC210	NA	TPYRTLLMNELGVPFHLG	P15
VIC210	NA	NELGVPFHLGTKQVCIAW	P15
VIC210	NA	LGTKQVCIAWSSSSCHDG	P15
VIC210	NA	AWSSSSCHDGKAWLHVCI	P16
VIC210	NA	DGKAWLHVCITGDDKNAT	P16
VIC210	NA	CITGDDKNATASFIYNGR	P16
VIC210	NA	ATASFIYNGRLVDSVVSW	P16
VIC210	NA	GRLVDSVVSWSKEILRTQ	P16
VIC210	NA	SWSKEILRTQESECVCIN	P16
VIC210	NA	TQESECVCINGTCTVVMT	P16
VIC210	NA	INGTCTVVMTDGSASGKA	P16
VIC210	NA	MTDGSASGKADTKILFIE	P16
VIC210	NA	KADTKILFIEEGKIVHTS	P16
VIC210	NA	IEEGKIVHTSTLSGSAQH	P16
VIC210	NA	TSTLSGSAQHVEECSCYPR	P16
VIC210	NA	QHVEECSCYPRYPGVRCV	P16
VIC210	NA	YPRYPGVRCVCRDNWKGS	P16
VIC210	NA	CVCRDNWKGSNRPIVDIN	P16
VIC210	NA	GSNRPIVDINIKDHSIVS	P16
VIC210	NA	INIKDHSIVSSYVCSGLV	P16
VIC210	NA	VSSYVCSGLVGDTPRKND	P16
VIC210	NA	LVGDTPRKNDSSSSSHCL	P16
VIC210	NA	NDSSSSSHCLDPNNEEGG	P16
VIC210	NA	CLDPNNEEGGHGVKGWAF	P16
VIC210	NA	GGHGVKGWAFDDGNDVWM	P16
VIC210	NA	AFDDGNDVWMGRTISEKS	P17
VIC210	NA	WMGRTISEKSRLGYETFK	P17
VIC210	NA	KSRLGYETFKVIEGWSNPK	P17
VIC210	NA	FKVIEGWSNPKSKLQINR	P17
VIC210	NA	NPKSKLQINRQVIVDRGN	P17
VIC210	NA	NRQVIVDRGNRSGYSGIFS	P17

VIC210	NA	GNRSGYSGIFSVEGKSCI	P17
VIC210	NA	IFSVEGKSCINRCFYVEL	P17
VIC210	NA	CINRCFYVELIRGRKEET	P17
VIC210	NA	ELIRGRKEETEVLWTSNS	P17
VIC210	NA	ETEVLWTSNSIVVFCGTS	P17
VIC210	NA	NSIVVFCGTSGTYGTGSW	P17
VIC210	NA	TSGTYGTGSWPDGADINL	P17
VIC210	NA	TYGTGSWPDGADINLMPI	P17
CA07	NP	MASQGTKRSYEQMETGGE	P18
CA07	NP	SYEQMETGGERQDATEIR	P18
CA07	NP	GERQDATEIRASVGRMIG	P18
CA07	NP	IRASVGRMIGGIGRFYIQ	P18
CA07	NP	IGGIGRFYIQMCTELKLS	P18
CA07	NP	IQMCTELKLSDYDGRLIQ	P18
CA07	NP	LSDYDGRLIQNSITIERM	P18
CA07	NP	IQNSITIERMVLSAFDER	P18
CA07	NP	RMVLSAFDERRNKYLEEH	P18
CA07	NP	YRRVDGKWMRELILYDKE	P18
CA07	NP	MRELILYDKEEIRRVWRQ	P18
CA07	NP	KEEIRRVWRQANNGEDAT	P18
CA07	NP	RQANNGEDATAGLTHIMI	P18
CA07	NP	ATAGLTHIMIWHSNLNDA	P18
CA07	NP	GSTLPRRSGAAGAAVKGV	P18
CA07	NP	GAAGAAVKGVGTIAMELI	P18
CA07	NP	GVGTIAMELIRMIKRGIN	P18
CA07	NP	LIRMIKRGINDRNFWRGE	P18
CA07	NP	INDRNFWRGENGRRTRVA	P18
CA07	NP	GENGRRTRVAYERMCNIL	P18
CA07	NP	VAYERMCNILKGKFQTAA	P18
CA07	NP	ILKGKFQTAAQRAMMDQV	P18
CA07	NP	AAQRAMMDQVRESRNPGN	P18
CA07	NP	ILRGSVAHKSCLPACVYG	P18
CA07	NP	KSCLPACVYGLAVASGHD	P19
CA07	NP	YGLAVASGHDFEREGYSL	P19
CA07	NP	HDFEREGYSLVGIDPFKL	P19
CA07	NP	SLVGIDPFKLLQNSQVVS	P19
CA07	NP	KLLQNSQVVSLMRPNENPA	P19
CA07	NP	VSLMRPNENPAHKSQLVW	P19
CA07	NP	VWMACHSAAFEDLRVSSF	P19
CA07	NP	AFEDLRVSSFIRGKKVIPR	P19
CA07	NP	SFIRGKKVIPRGKLSTRG	P19
CA07	NP	IPRGKLSTRGVQIASNEN	P19
CA07	NP	RGVQIASNENVETMDSNT	P19
CA07	NP	ENVETMDSNTLELRSRYW	P19
CA07	NP	NTLELRSRYWAIRTRSGG	P19
CA07	NP	YWAIRTRSGGNTNQQKAS	P19
CA07	NP	GGNTNQQKASAGQISVQPT	P19
CA07	NP	ASAGQISVQPTFSVQRNL	P19
CA07	NP	QPTFSVQRNLPFERATVM	P19
CA07	NP	NLPFERATVMAAFSGNNE	P19
CA07	NP	VMAAFSGNNEGRTSDMRT	P19
CA07	NP	NEGRTSDMRTEVIRMMES	P19
CA07	NP	RTEVIRMMESAKPEDLSF	P19
CA07	NP	ESAKPEDLSFQGRGVFEL	P19

CA07	NP	SFQGRGVFELSDEKATNPI	P19
CA07	NP	MSNEGSYFFGDNAEEYDS	P19
VIC210	NP	MASQGTKRSYEQMETDGD	P20
VIC210	NP	SYEQMETDGDRQNATEIR	P20
VIC210	NP	GDRQNATEIRASVGKMID	P20
VIC210	NP	IRASVGKMIDGIGRFYIQ	P20
VIC210	NP	IDGIGRFYIQMCTELKLS	P20
VIC210	NP	IQMCTELKLSDHEGRLIQ	P20
VIC210	NP	LSDHEGRLIQNSLTIEKM	P20
VIC210	NP	IQNSLTIEKMVLSAFDER	P20
VIC210	NP	KMVLSAFDERRNKYLEEH	P20
VIC210	NP	YRRVDGKWMRELVLYDKE	P20
VIC210	NP	MRELVLYDKEEIRRIWRQ	P20
VIC210	NP	KEEIRRIWRQANNGEDAT	P20
VIC210	NP	RQANNGEDATSGLTHIMI	P20
VIC210	NP	ATSGLTHIMIWHSNLNDA	P20
VIC210	NP	GSTLPRRSGAAGAAVKGI	P20
VIC210	NP	GAAGAAVKGIGTMVMELI	P20
VIC210	NP	GIGTMVMELIRMVKRGIN	P20
VIC210	NP	LIRMVKRGINDRNFWRGE	P20
VIC210	NP	INDRNFWRGENGRKTRSA	P20
VIC210	NP	GENGRKTRSAYERMCNIL	P20
VIC210	NP	SAYERMCNILKGKFQTAA	P20
VIC210	NP	ILKGKFQTAAQRAMVDQV	P20
VIC210	NP	AAQRAMVDQVRESRNPGN	P20
VIC210	NP	ILRGSVAHKSCLPACAYG	P20
VIC210	NP	KSCLPACAYGPAVSSGYD	P21
VIC210	NP	YGPAVSSGYDFEKEGYSL	P21
VIC210	NP	YDFEKEGYSLVGIDPFKL	P21
VIC210	NP	SLVGIDPFKLLQNSQIYS	P21
VIC210	NP	KLLQNSQIYSLIRPNENPA	P21
VIC210	NP	YSLIRPNENPAHKSQLVW	P21
VIC210	NP	VWMACHSAAFEDLRLLSF	P21
VIC210	NP	AFEDLRLLSFIRGTKVSPR	P21
VIC210	NP	SFIRGTKVSPRGKLSTRG	P21
VIC210	NP	SPRGKLSTRGVQIASNEN	P21
VIC210	NP	RGVQIASNENMDNMGSST	P21
VIC210	NP	ENMDNMGSSTLELRSGYW	P21
VIC210	NP	STLELRSGYWAIRTRSGG	P21
VIC210	NP	YWAIRTRSGGNTNQQRAS	P21
VIC210	NP	GGNTNQQRASAGQTSVQPT	P21
VIC210	NP	ASAGQTSVQPTFSVQRNL	P21
VIC210	NP	QPTFSVQRNLPFEKSTIM	P21
VIC210	NP	NLPFEKSTIMAAFTGNTE	P21
VIC210	NP	IMAAFTGNTEGRTSDMRA	P21
VIC210	NP	TEGRTSDMRAEIIRMMES	P21
VIC210	NP	RAEIIRMMESAKPEEVSF	P21
VIC210	NP	ESAKPEEVSFRGRGVFEL	P21
VIC210	NP	SFRGRGVFELSDEKATNPI	P21
VIC210	NP	MSNEGSYFFGDNAEEYDN	P21
CA07/VIC210	NP	ERRNKYLEEHPSAGKDPK	P22
CA07/VIC210	NP	EHPSAGKDPKKTGGPIYR	P22
CA07/VIC210	NP	PKKTGGPIYRRVDGKWMR	P22
CA07/VIC210	NP	MIWHSNLNDATYQRTRAL	P22

CA07/VIC210	NP	DATYQRTRALVRTGMDPR	P22
CA07/VIC210	NP	ALVRTGMDPRMCSLMQGS	P22
CA07/VIC210	NP	PRMCSLMQGSTLPRRSGA	P22
CA07/VIC210	NP	QVRESRNPGNAEIEDLIF	P22
CA07/VIC210	NP	GNAEIEDLIFLARSALIL	P22
CA07/VIC210	NP	IFLARSALILRGSVAHKS	P22
CA07/VIC210	NP	NPAHKSQLVWMACHSAAF	P22
CA07/VIC210	NP	ELSDEKATNPIVPSFDMS	P22
CA07/VIC210	NP	NPIVPSFDMSNEGSYFFG	P22
CA07	NS1	MDSNTMSSFQVDCFLWHI	P23
CA07	NS1	FQVDCFLWHIRKRFADNG	P23
CA07	NS1	HIRKRFADNGLGDAPFLD	P23
CA07	NS1	NGLGDAPFLDRLRRDQKS	P23
CA07	NS1	LDRLRRDQKSLKGRGNTL	P23
CA07	NS1	KSLKGRGNTLGLDIETAT	P23
CA07	NS1	TLGLDIETATLVGKQIVE	P23
CA07	NS1	ATLVGKQIVEWILKEESS	P23
CA07	NS1	VEWILKEESSETLRMTIA	P23
CA07	NS1	SSETLRMTIASVPTSRYL	P23
CA07	NS1	IASVPTSRYLSDMTLEEM	P23
CA07	NS1	YLSDMTLEEMSRDWFMLM	P23
CA07	NS1	EMSRDWFMLMPRQKIIGPL	P23
CA07	NS1	LMPRQKIIGPLCVRLDQA	P23
CA07	NS1	GPLCVRLDQAIMEKNIVL	P24
CA07	NS1	QAIMEKNIVLKANFSVIF	P24
CA07	NS1	VLKANFSVIFNRLETLIL	P24
CA07	NS1	IFNRLETLILLRAFTEEG	P24
CA07	NS1	ILLRAFTEEGAIVGEISPL	P24
CA07	NS1	EGAIVGEISPLPSLPGHT	P24
CA07	NS1	SPLPSLPGHTYEDVKNAV	P24
CA07	NS1	HTYEDVKNAVGVLIGGLE	P24
CA07	NS1	AVGVLIGGLEWNGNTVRV	P24
CA07	NS1	LEWNGNTVRVSENIQRFA	P24
CA07	NS1	RVSENIQRFAWRNCDENG	P24
CA07	NS1	FAWRNCDENGRPSLPPEQ	P24
CA07	NS1	AWRNCDENGRPSLPPEQK	P24
VIC210	NS1	MDSNTVSSFQVDCFLWHI	P25
VIC210	NS1	FQVDCFLWHIRKQVVDQE	P25
VIC210	NS1	HIRKQVVDQELSDAPFLD	P25
VIC210	NS1	QELSDAPFLDRLRRDQRS	P25
VIC210	NS1	LDRLRRDQRSLRGRGNTL	P25
VIC210	NS1	RSLRGRGNTLGLDIKAAT	P25
VIC210	NS1	TLGLDIKAATHVGKQIVE	P25
VIC210	NS1	ATHVGKQIVEKILKEESD	P25
VIC210	NS1	VEKILKEESDEALKMTMV	P25
VIC210	NS1	SDEALKMTMVSTPASRYI	P25
VIC210	NS1	MVSTPASRYITDMTIEEL	P25
VIC210	NS1	YITDMTIEELSRNWFMLM	P25
VIC210	NS1	ELSRNWFMLMPKQKMEGPL	P25
VIC210	NS1	LMPKQKMEGPLCIRMDQA	P25
VIC210	NS1	GPLCIRMDQAIMEKNIML	P26
VIC210	NS1	QAIMEKNIMLKANFSVIF	P26
VIC210	NS1	MLKANFSVIFDRLETIVL	P26
VIC210	NS1	IFDRLETIVLLRAFTEEG	P26

VIC210	NS1	VLLRAFTEEGAIVGEISPL	P26
VIC210	NS1	EGAIVGEISPLPSFPGHT	P26
VIC210	NS1	SPLPSFPGHTIEDVKNAI	P26
VIC210	NS1	HTIEDVKNAIGVLIGGLE	P26
VIC210	NS1	AIGVLIGGLEWNDNTVRV	P26
VIC210	NS1	LEWNDNTVRVSKNLQRFA	P26
VIC210	NS1	RVSKNLQRFAWRSSNENG	P26
VIC210	NS1	FAWRSSNENGGSPLTPKQ	P26
VIC210	NS1	NGGSPLTPKQKREMARTA	P26
VIC210	NS1	PLTPKQKREMARTARSKV	P26
CA07	NS2	MDSNTMSSFQDILMRMSK	P27
CA07	NS2	FQDILMRMSKMQLGSSSE	P27
CA07	NS2	SKMQLGSSSEDLNGMVTR	P27
CA07	NS2	SEDLNGMVTRFESLKIYR	P27
CA07	NS2	TRFESLKIYRDSLGETVM	P27
CA07	NS2	YRDSLGETVMRMGDLHYL	P27
CA07	NS2	VMRMGDLHYLQSRNEKWR	P27
CA07	NS2	YLQSRNEKWREQLGQKFE	P27
CA07	NS2	FEEIRWLIEEMRHRLKAT	P27
CA07	NS2	EEMRHRLKATENSFEQIT	P27
CA07	NS2	ATENSFEQITFMQALQLL	P27
CA07	NS2	ITFMQALQLLLEVEQEIR	P27
CA07	NS2	QLLLEVEQEIRAFSFQLI	P27
VIC210	NS2	MDSNTVSSFQDILLRMSK	P28
VIC210	NS2	FQDILLRMSKMQLGSSSE	P28
VIC210	NS2	SKMQLGSSSEDLNGMITQ	P28
VIC210	NS2	SEDLNGMITQFESLKIYR	P28
VIC210	NS2	TQFESLKIYRDSLGEAVM	P28
VIC210	NS2	YRDSLGEAVMRMGDLHLL	P28
VIC210	NS2	VMRMGDLHLLQNRNGKWR	P28
VIC210	NS2	LLQNRNGKWREQLGQKFE	P28
VIC210	NS2	FEEIRWLIEEVRHRLKTT	P28
VIC210	NS2	EEVRHRLKTTENSFEQIT	P28
VIC210	NS2	TTENSFEQITFMQALQLL	P28
VIC210	NS2	ITFMQALQLLFEVEQEIR	P28
VIC210	NS2	QLLFEVEQEIRTFSFQLI	P28
CA07/VIC210	NS2	WREQLGQKFEEIRWLIEE	P28
CA07	PA	AEKAMKEYGEDPKIETNK	P29
CA07	PA	GEDPKIETNKFAAICTHL	P29
CA07	PA	HLEVCFMYSDFHFIDERG	P29
CA07	PA	SDFHFIDERGESIIVESG	P29
CA07	PA	RGESIIVESGDPNALLKH	P29
CA07	PA	SGDPNALLKHRFEIIEGR	P29
CA07	PA	KHRFEIIEGRDRIMAWTV	P29
CA07	PA	GRDRIMAWTVVNSICNTT	P29
CA07	PA	TVVNSICNTTGVEKPKFL	P29
CA07	PA	TTGVEKPKFLPDLYDYKE	P29
CA07	PA	IYYLEKANKIKSEKTHIH	P29
CA07	PA	KIKSEKTHIHIFSFTGEE	P29
CA07	PA	ARIKTRLFTIRQEMASRS	P29
CA07	PA	TIRQEMASRSLWDSFRQS	P29
CA07	PA	RSLWDSFRQSERGEETIE	P29
CA07	PA	IEEKFEITGTMRKLADQS	P29
CA07	PA	GTMRKLADQSLPPNFPSL	P29

CA07	PA	QSLPPNFPSLENFRAYVD	P29
CA07	PA	SLENFRAYVDGFEPNGCI	P29
CA07	PA	CIEGKLSQMSKEVNAKIE	P29
CA07	PA	MSKEVNAKIEPFLRTTPR	P29
CA07	PA	IEPFLRTTPRPLRLPDGPL	P29
CA07	PA	PRPLRLPDGPLCHQRSKF	P30
CA07	PA	LQDIENEEKIPRTKNMKR	P30
CA07	PA	KIPRTKNMKRTSQLKWAL	P30
CA07	PA	KRTSQLKWALGENMAPEK	P30
CA07	PA	ALGENMAPEKVDFDDCKD	P30
CA07	PA	EKVDFDDCKDVGDLKQYD	P30
CA07	PA	KDVGDLKQYDSDEPEPRS	P30
CA07	PA	YDSDEPEPRSLASWVQNE	P30
CA07	PA	RSLASWVQNEFNKACELT	P30
CA07	PA	NEFNKACELTDSSWIELD	P30
CA07	PA	LTDSSWIELDEIGEDVAPI	P30
CA07	PA	WEKYCVLEIGDMLLRTAI	P30
CA07	PA	IGDMLLRTAIGQVSRPMF	P30
CA07	PA	AIGQVSRPMFLYVRTNGT	P30
CA07	PA	MFLYVRTNGTSKIKMKWG	P30
CA07	PA	GTSKIKMKWGMEMRRCLL	P30
CA07	PA	SVKEKDMTKEFFENKSET	P30
CA07	PA	KEFFENKSETWPIGESPR	P30
CA07	PA	ETWPIGESPRGVEEGSIG	P30
CA07	PA	PRGVEEGSIGKVCRTLLA	P30
CA07	PA	GFSAESRKLLLIVQALRD	P30
CA07	PA	LLLIVQALRDNLEPGTFD	P30
VIC210	PA	AEKAMKEYGEDLKIETNK	P31
VIC210	PA	GEDLKIETNKFAAICTHL	P31
VIC210	PA	HLEVCFMYSDFHFINEQG	P31
VIC210	PA	SDFHFINEQGESIVVELD	P31
VIC210	PA	QGESIVVELDDPNALLKH	P31
VIC210	PA	LDDPNALLKHRFEIIEGR	P31
VIC210	PA	KHRFEIIEGRDRTMAWTV	P31
VIC210	PA	GRDRTMAWTVVNSICNTT	P31
VIC210	PA	TVVNSICNTTGAGKPKFL	P31
VIC210	PA	TTGAGKPKFLPDLYDYKE	P31
VIC210	PA	IYYLEKANKIKSENTHIH	P31
VIC210	PA	KIKSENTHIHIFSFTGEE	P31
VIC210	PA	ARIKTRLFTIRQEMANRG	P31
VIC210	PA	TIRQEMANRGLWDSFRQS	P31
VIC210	PA	RGLWDSFRQSERGEETIE	P31
VIC210	PA	IEEKFEITGTMRRLADQS	P31
VIC210	PA	GTMRRLADQSLPPNFSCL	P31
VIC210	PA	QSLPPNFSCLENFRAYVD	P31
VIC210	PA	CLENFRAYVDGFEPNGCI	P31
VIC210	PA	CIEGKLSQMSKEVNAQIE	P31
VIC210	PA	MSKEVNAQIEPFLKTTPR	P31
VIC210	PA	IEPFLKTTPRPIKLPNGPPC	P31
VIC210	PA	PRPIKLPNGPPCYQRSKF	P32
VIC210	PA	XQDVENEEKVPRTKNMKK	P32
VIC210	PA	KVPRTKNMKKTSQLKWAL	P32
VIC210	PA	KKTSQLKWALGENMAPEK	P32
VIC210	PA	ALGENMAPEKVDFENCRD	P32

VIC210	PA	EKVDFENCRDISDLKQYD	P32
VIC210	PA	RDISDLKQYDSDEPELRS	P32
VIC210	PA	YDSDEPELRSLSSWIQNE	P32
VIC210	PA	RSLSSWIQNEFNKACELT	P32
VIC210	PA	NEFNKACELTDSVWIELD	P32
VIC210	PA	LTDSVWIELDEIGEDVAPI	P32
VIC210	PA	WEKYCVLEIGDMLLRSAI	P32
VIC210	PA	IGDMLLRSAIGQISRPMF	P32
VIC210	PA	AIGQISRPMFLYVRTNGT	P32
VIC210	PA	MFLYVRTNGTSKVKMKWG	P32
VIC210	PA	GTSKVKMKWGMEMRRCLL	P32
VIC210	PA	SVKEKDMTKEFFENKSEA	P32
VIC210	PA	KEFFENKSEAWPIGESPK	P32
VIC210	PA	EAWPIGESPKGVEEGSIG	P32
VIC210	PA	PKGVEEGSIGKVCRTLLA	P32
VIC210	PA	GFSAESRKLLLVVQALRD	P32
VIC210	PA	LLLVVQALRDNLEPGTFD	P32
CA07	PA	GPLCHQRSKFLLMDALKL	P33
CA07	PA	KFLLMDALKLSIEDPSHE	P33
CA07	PA	KLSIEDPSHEGEGIPLYD	P33
CA07	PA	HEGEGIPLYDAIKCMKTF	P33
CA07	PA	YDAIKCMKTFFGWKEPNI	P33
CA07	PA	TFFGWKEPNIVKPHEKGI	P33
CA07	PA	NIVKPHEKGINPNYLMAW	P33
CA07	PA	GINPNYLMAWKQVLAELQ	P33
CA07	PA	AWKQVLAELQDIENEEKI	P33
CA07/VIC210	PA	MEDFVRQCFNPMIVELAE	P34
CA07/VIC210	PA	FNPMIVELAEKAMKEYGE	P34
CA07/VIC210	PA	NKFAAICTHLEVCFMYSD	P34
CA07/VIC210	PA	FLPDLYDYKENRFIEIGV	P34
CA07/VIC210	PA	KENRFIEIGVTRREVHIY	P34
CA07/VIC210	PA	GVTRREVHIYYLEKANKI	P34
CA07/VIC210	PA	IHIFSFTGEEMATKADYT	P34
CA07/VIC210	PA	EEMATKADYTLDEESRAR	P34
CA07/VIC210	PA	YTLDEESRARIKTRLFTI	P34
CA07/VIC210	PA	QSERGEETIEEKFEITGT	P34
CA07/VIC210	PA	VDGFEPNGCIEGKLSQMS	P34
CA07/VIC210	PA	LDEIGEDVAPIEHIASMR	P34
CA07/VIC210	PA	APIEHIASMRRNYFTAEV	P34
CA07/VIC210	PA	MRRNYFTAEVSHCRATEY	P34
CA07/VIC210	PA	EVSHCRATEYIMKGVYIN	P34
CA07/VIC210	PA	EYIMKGVYINTALLNASC	P34
CA07/VIC210	PA	INTALLNASCAAMDDFQL	P34
CA07/VIC210	PA	SCAAMDDFQLIPMISKCR	P34
CA07/VIC210	PA	QLIPMISKCRTKEGRRKT	P34
CA07/VIC210	PA	CRTKEGRRKTNLYGFIIK	P34
CA07/VIC210	PA	KTNLYGFIIKGRSHLRND	P34
CA07/VIC210	PA	IKGRSHLRNDTDVVNFVS	P34
CA07/VIC210	PA	NDTDVVNFVSMEFSLTDPR	P35
CA07/VIC210	PA	VSMEFSLTDPRLEPHKWE	P35
CA07/VIC210	PA	DPRLEPHKWEKYCVLEIG	P35
CA07/VIC210	PA	WGMEMRRCLLQSLQQIES	P35
CA07/VIC210	PA	LLQSLQQIESMIEAESSV	P35
CA07/VIC210	PA	ESMIEAESSVKEKDMTKE	P35

CA07/VIC210	PA	IGKVCRTLLAKSVFNSLY	P35
CA07/VIC210	PA	LAKSVFNSLYASPQLEGF	P35
CA07/VIC210	PA	LYASPQLEGFSAESRKLL	P35
CA07/VIC210	PA	RDNLEPGTFDLGGLYEAI	P35
CA07/VIC210	PA	FDLGGLYEAIEECLINDPW	P35
CA07/VIC210	PA	AIEECLINDPWVLLNASW	P35
CA07/VIC210	PA	DPWVLLNASWFNSFLTHA	P35
CA07/VIC210	PA	WVLLNASWFNSFLTHALK	P35
CA07	PB1	MDVNPTLLFLKIPAQNAI	P36
CA07	PB1	FLKIPAQNAISTTFPYTG	P36
CA07	PB1	DTVNRTHQYSEKGKWTTN	P36
CA07	PB1	YSEKGKWTTNTETGAPQL	P36
CA07	PB1	ESHPGIFENSCLETMEVV	P36
CA07	PB1	NSCLETMEVVQQTRVDKL	P36
CA07	PB1	VVQQTRVDKLTQGRQTYD	P36
CA07	PB1	GRLIDFLKDVMESMNKEE	P36
CA07	PB1	DVMESMNKEEIEITTHFQ	P36
CA07	PB1	EEIEITTHFQRKRRVRDN	P36
CA07	PB1	TQRTIGKKKQRLNKRGYL	P36
CA07	PB1	KQRLNKRGYLIRALTLNT	P36
CA07	PB1	FSMELPSFGVSGVNESAD	P36
CA07	PB1	GVSGVNESADMSIGVTVI	P36
CA07	PB1	CHRGDTQIQTRRSFELKK	P37
CA07	PB1	QTRRSFELKKLWDQTQSK	P37
CA07	PB1	KKLWDQTQSKVGLLVSDG	P37
CA07	PB1	SKVGLLVSDGGPNLYNIR	P37
CA07	PB1	IRNLHIPEVCLKWELMDD	P37
CA07	PB1	VCLKWELMDDDYRGRLCN	P37
CA07	PB1	DDDYRGRLCNPLNPFVSH	P37
CA07	PB1	CNPLNPFVSHKEIDSVNN	P37
CA07	PB1	SHKEIDSVNNAVVMPAHG	P37
CA07	PB1	EKFFPSSSYRRPVGISSM	P37
CA07	PB1	YRRPVGISSMVEAMVSRA	P37
CA07	PB1	SMVEAMVSRARIDARVDF	P37
CA07	PB1	RARIDARVDFESGRIKKE	P37
VIC210	PB1	MDVNPTLLFLKVPAQNAI	P38
VIC210	PB1	FLKVPAQNAISTTFPYTG	P38
VIC210	PB1	DTVNRTHQYSERGKWTTN	P38
VIC210	PB1	YSERGKWTTNTETGAPQL	P38
VIC210	PB1	ESHPGIFENSCLETMEAV	P38
VIC210	PB1	NSCLETMEAVQQTRVDKL	P38
VIC210	PB1	AVQQTRVDKLTQGRQTYD	P38
VIC210	PB1	GRLIDFLKDVMESMDKEE	P38
VIC210	PB1	DVMESMDKEEMEITTHFQ	P38
VIC210	PB1	EEMEITTHFQRKRRVRDN	P38
VIC210	PB1	TQRTIGKKKQRVNKRGYL	P38
VIC210	PB1	KQRVNKRGYLIRALTLNT	P38
VIC210	PB1	FSMELPSFGVSGINESAD	P38
VIC210	PB1	GVSGINESADMSIGVTVI	P38
VIC210	PB1	CHRGDTQIQTRRSFEIKK	P39
VIC210	PB1	QTRRSFEIKKLWDQTQSR	P39
VIC210	PB1	KKLWDQTQSRAGLLVSDG	P39
VIC210	PB1	SRAGLLVSDGGPNLYNIR	P39
VIC210	PB1	IRNLHIPEVCLKWELMDE	P39

VIC210	PB1	VCLKWELMDENYRGRLCN	P39
VIC210	PB1	DENYRGRLCNPLNPFVSH	P39
VIC210	PB1	CNPLNPFVSHKEIESVNN	P39
VIC210	PB1	SHKEIESVNNAVVMPAHG	P39
VIC210	PB1	EKFFPSSSYRRPIGISSM	P39
VIC210	PB1	YRRPIGISSMVEAMVSRA	P39
VIC210	PB1	SMVEAMVSRARIDARIDF	P39
VIC210	PB1	RARIDARIDFESGRIKKE	P39
CA07	PB1	IATPGMQIRGFVYFVETL	P40
CA07	PB1	RGFVYFVETLARSICEKL	P40
CA07	PB1	TLARSICEKLEQSGLPVG	P40
CA07	PB1	KLEQSGLPVGGNEKKAKL	P40
CA07	PB1	VGGNEKKAKLANVVRKMM	P40
CA07	PB1	KLANVVRKMMTNSQDTEI	P40
CA07	PB1	MMTNSQDTEISFTITGDN	P40
CA07	PB1	EISFTITGDNTKWNENQN	P40
CA07	PB1	DNTKWNENQNPRMFLAMI	P40
CA07	PB1	QNPRMFLAMITYITRNQPE	P40
CA07	PB1	MITYITRNQPEWFRNILS	P40
CA07	PB1	QPEWFRNILSMAPIMFSN	P40
CA07	PB1	LSMAPIMFSNKMARLGKG	P40
CA07	PB1	SNKMARLGKGYMFESKRM	P40
CA07	PB1	KGYMFESKRMKIRTQIPA	P41
CA07	PB1	RMKIRTQIPAEMLASIDL	P41
CA07	PB1	PAEMLASIDLKYFNESTK	P41
CA07	PB1	DLKYFNESTKKKIEKIRPL	P41
CA07	PB1	TKKKIEKIRPLLIDGTAS	P41
CA07	PB1	RPLLIDGTASLSPGMMMG	P41
CA07	PB1	ASLSPGMMMGMFNMLSTV	P41
CA07	PB1	MGMFNMLSTVLGVSILNL	P41
CA07	PB1	TVLGVSILNLGQKKYTKT	P41
CA07	PB1	NLGQKKYTKTIYWWDGLQ	P41
CA07	PB1	KTIYWWDGLQSSDDFALI	P41
CA07	PB1	LQSSDDFALIVNAPNHEG	P41
CA07	PB1	LIVNAPNHEGIQAGVDRF	P41
CA07	PB1	EGIQAGVDRFYRTCKLVG	P41
CA07/VIC210	PB1	AISTTFPYTGDPPYSHGT	P42
CA07/VIC210	PB1	TGDPPYSHGTGTGYTMDT	P42
CA07/VIC210	PB1	GTGTGYTMDTVNRTHQYS	P42
CA07/VIC210	PB1	TNTETGAPQLNPIDGPLPE	P42
CA07/VIC210	PB1	QLNPIDGPLPEDNEPSGY	P42
CA07/VIC210	PB1	LPEDNEPSGYAQTDCVLE	P42
CA07/VIC210	PB1	GYAQTDCVLEAMAFLEES	P42
CA07/VIC210	PB1	LEAMAFLEESHPGIFENS	P42
CA07/VIC210	PB1	KLTQGRQTYDWTLNRNQPA	P42
CA07/VIC210	PB1	YDWTLNRNQPAATALANT	P42
CA07/VIC210	PB1	QPAATALANTIEVFRSNG	P42
CA07/VIC210	PB1	NTIEVFRSNGLTANESGR	P42
CA07/VIC210	PB1	NGLTANESGRLIDFLKDV	P42
CA07/VIC210	PB1	FQRKRRVRDNMTKKMVTQ	P42
CA07/VIC210	PB1	DNMTKKMVTQRTIGKKKQ	P42
CA07/VIC210	PB1	YLIRALTLNTMTKDAERG	P42
CA07/VIC210	PB1	NTMTKDAERGKLKRRAIA	P42
CA07/VIC210	PB1	RGKLKRRAIATPGMQIRG	P42

CA07/VIC210	PB1	RFYRTCKLVGINMSKKKS	P42
CA07/VIC210	PB1	VGINMSKKKSYINKTGTF	P42
CA07/VIC210	PB1	KSYINKTGTFEFTSFFYR	P43
CA07/VIC210	PB1	TFEFTSFFYRYGFVANFS	P43
CA07/VIC210	PB1	YRYGFVANFSMELPSFGV	P43
CA07/VIC210	PB1	ADMSIGVTVIKNNMINND	P43
CA07/VIC210	PB1	VIKNNMINNDLGPATAQM	P43
CA07/VIC210	PB1	NDLGPATAQMALQLFIKD	P43
CA07/VIC210	PB1	QMALQLFIKDYRYTYRCH	P43
CA07/VIC210	PB1	KDYRYTYRCHRGDTQIQT	P43
CA07/VIC210	PB1	DGGPNLYNIRNLHIPEVC	P43
CA07/VIC210	PB1	NNAVVMPAHGPAKSMEYD	P43
CA07/VIC210	PB1	HGPAKSMEYDAVATTHSW	P43
CA07/VIC210	PB1	YDAVATTHSWIPKRNRSI	P43
CA07/VIC210	PB1	SWIPKRNRSILNTSQRGI	P43
CA07/VIC210	PB1	SILNTSQRGILEDEQMYQ	P43
CA07/VIC210	PB1	GILEDEQMYQKCCNLFEK	P43
CA07/VIC210	PB1	YQKCCNLFEKFFPSSSYR	P43
CA07/VIC210	PB1	DFESGRIKKEEFSEIMKI	P43
CA07/VIC210	PB1	KEEFSEIMKICSTIEELR	P43
CA07/VIC210	PB1	FSEIMKICSTIEELRRQK	P43
CA07	PB2	MERIKELRDLMSQSRTRE	P44
CA07	PB2	DLMSQSRTREILTKTTVD	P44
CA07	PB2	KYTSGRQEKNPALRMKWM	P44
CA07	PB2	KNPALRMKWMMAMRYPIT	P44
CA07	PB2	WMMAMRYPITADKRIMDM	P44
CA07	PB2	ITADKRIMDMIPERNEQG	P44
CA07	PB2	DMIPERNEQGQTLWSKTN	P44
CA07	PB2	QGQTLWSKTNDAGSDRVM	P44
CA07	PB2	TNDAGSDRVMVSPLAVTW	P44
CA07	PB2	VMVSPLAVTWWNRNGPTT	P44
CA07	PB2	TWWNRNGPTTSTVHYPKV	P44
CA07	PB2	TTSTVHYPKVYKTYFEKV	P44
CA07	PB2	KVYKTYFEKVERLKHGTF	P44
CA07	PB2	NQVKIRRRVDTNPGHADL	P44
CA07	PB2	VDTNPGHADLSAKEAQDV	P44
CA07	PB2	PNEVGARILTSESQLAIT	P44
CA07	PB2	LTSESQLAITKEKKEELQ	P44
CA07	PB2	ITKEKKEELQDCKIAPLM	P44
CA07	PB2	LQDCKIAPLMVAYMLERE	P44
CA07	PB2	LSPEEVSETQGTEKLTIT	P44
CA07	PB2	TQGTEKLTITYSSSMMWE	P45
CA07	PB2	VLVNTYQWIIRNWEIVKI	P45
CA07	PB2	IIRNWEIVKIQWSQDPTM	P45
CA07	PB2	KIQWSQDPTMLYNKMEFE	P45
CA07	PB2	TMLYNKMEFEPFQSLVPK	P45
CA07	PB2	FEPFQSLVPKATRSRYSG	P45
CA07	PB2	PKATRSRYSGFVRTLFQQ	P45
CA07	PB2	QQMRDVLGTFDTVQIIKL	P45
CA07	PB2	TFDTVQIIKLLPFAAAPPE	P45
CA07	PB2	KLLPFAAAPPEQSRMQFS	P45
CA07	PB2	PPEQSRMQFSSLTVNVRG	P45
CA07	PB2	FSSLTVNVRGSGLRILVR	P45
CA07	PB2	RGSGLRILVRGNSPVFNY	P45

CA07	PB2	VRGNSPVFNYNKATKRLT	P45
CA07	PB2	NYNKATKRLTVLGKDAGA	P45
CA07	PB2	LTVLGKDAGALTEDPDEG	P45
CA07	PB2	GALTEDPDEGTSGVESAV	P45
CA07	PB2	EGTSGVESAVLRGFLILG	P45
CA07	PB2	AVLRGFLILGKEDKRYGPA	P45
CA07	PB2	LGKEDKRYGPALSINELS	P45
VIC210	PB2	MERIKELRNLMSQSRTRE	P46
VIC210	PB2	NLMSQSRTREILTKTTVD	P46
VIC210	PB2	KYTSGRQEKNPSLRMKWM	P46
VIC210	PB2	KNPSLRMKWMMAMKYPIT	P46
VIC210	PB2	WMMAMKYPITADKRITEM	P46
VIC210	PB2	ITADKRITEMVPERNEQG	P46
VIC210	PB2	EMVPERNEQGQTLWSKMS	P46
VIC210	PB2	QGQTLWSKMSDAGSDRVM	P46
VIC210	PB2	MSDAGSDRVMVSPLAVTW	P46
VIC210	PB2	VMVSPLAVTWWNRNGPVT	P46
VIC210	PB2	TWWNRNGPVTSTVHYPKV	P46
VIC210	PB2	VTSTVHYPKVYKTYFDKV	P46
VIC210	PB2	KVYKTYFDKVERLKHGTF	P46
VIC210	PB2	NQVKIRRRVDINPGHADL	P46
VIC210	PB2	VDINPGHADLSAKEAQDV	P46
VIC210	PB2	PNEVGARILTSESQLTIT	P46
VIC210	PB2	LTSESQLTITKEKKEELR	P46
VIC210	PB2	ITKEKKEELRDCKISPLM	P46
VIC210	PB2	LRDCKISPLMVAYMLERE	P46
VIC210	PB2	LSPEEVSETQGTERLTIT	P46
VIC210	PB2	TQGTERLTITYSSSMMWE	P47
VIC210	PB2	VLVNTYQWIIRNWEAVKI	P47
VIC210	PB2	IIRNWEAVKIQWSQNPAM	P47
VIC210	PB2	KIQWSQNPAMLYNKMEFE	P47
VIC210	PB2	AMLYNKMEFEPFQSLVPK	P47
VIC210	PB2	FEPFQSLVPKAIRSQYSG	P47
VIC210	PB2	PKAIRSQYSGFVRTLFQQ	P47
VIC210	PB2	QQMRDVLGTFDTTQIIKL	P47
VIC210	PB2	TFDTTQIIKLLPFAAAPPK	P47
VIC210	PB2	KLLPFAAAPPKQSRMQFS	P47
VIC210	PB2	PPKQSRMQFSSLTVNVRG	P47
VIC210	PB2	FSSLTVNVRGSGMRILVR	P47
VIC210	PB2	RGSGMRILVRGNSPVFNY	P47
VIC210	PB2	VRGNSPVFNYNKTTKRLT	P47
VIC210	PB2	NYNKTTKRLTILGKDAGT	P47
VIC210	PB2	LTILGKDAGTLIEDPDES	P47
VIC210	PB2	GTLIEDPDESTSGVESAV	P47
VIC210	PB2	ESTSGVESAVLRGFLIIG	P47
VIC210	PB2	AVLRGFLIIGKEDRRYGPA	P47
VIC210	PB2	IGKEDRRYGPALSINELS	P47
CA07	PB2	RELVRKTRFLPVAGGTGS	P48
CA07	PB2	FLPVAGGTGSVYIEVLHL	P48
CA07	PB2	GSVYIEVLHLTQGTCWEQ	P48
CA07	PB2	HLTQGTCWEQMYTPGGEV	P48
CA07	PB2	EQMYTPGGEVRNDDVDQS	P48
CA07	PB2	EVRNDDVDQSLIIAARNI	P48
CA07	PB2	QSLIIAARNIVRRAAVSA	P48

CA07	PB2	NIVRRAAVSADPLASLLE	P48
CA07	PB2	SADPLASLLEMCHSTQIG	P48
CA07	PB2	LEMCHSTQIGGVRMVDIL	P48
CA07	PB2	IGGVRMVDILRQNPTEEQ	P48
CA07	PB2	ILRQNPTEEQAVDICKAA	P48
CA07	PB2	EQAVDICKAAIGLRISSS	P48
CA07	PB2	AAIGLRISSSFSFGGFTF	P48
CA07	PB2	SSFSFGGFTFKRTSGSSV	P48
CA07	PB2	TFKRTSGSSVKKEEEVLT	P48
CA07	PB2	SVKKEEEVLTGNLQTLKI	P48
CA07	PB2	LTGNLQTLKIRVHEGYEE	P48
CA07	PB2	KIRVHEGYEEFTMVGRRA	P48
CA07	PB2	EEFTMVGRRATAILRKAT	P48
CA07	PB2	RATAILRKATRRLIQLIV	P49
CA07	PB2	ATRRLIQLIVSGRDEQSI	P49
CA07	PB2	IVSGRDEQSIAEAIIVAM	P49
CA07	PB2	SIAEAIIVAMVFSQEDCM	P49
CA07	PB2	AMVFSQEDCMIKAVRGDL	P49
CA07	PB2	CMIKAVRGDLNFVNRANQ	P49
CA07	PB2	DLNFVNRANQRLNPMHQL	P49
CA07	PB2	NQRLNPMHQLLRHFQKDA	P49
CA07	PB2	QLLRHFQKDAKVLFQNWG	P49
CA07	PB2	DAKVLFQNWGIESIDNVM	P49
CA07	PB2	WGIESIDNVMGMIGILPD	P49
CA07	PB2	VMGMIGILPDMTPSTEMS	P49
CA07	PB2	PDMTPSTEMSLRGIRVSK	P49
CA07	PB2	MSLRGIRVSKMGVDEYSS	P49
CA07	PB2	SKMGVDEYSSTERVVVSI	P49
CA07	PB2	SSTERVVVSIDRFLRVRD	P49
CA07/VIC210	PB2	REILTKTTVDHMAIIKKY	P50
CA07/VIC210	PB2	VDHMAIIKKYTSGRQEKN	P50
CA07/VIC210	PB2	KVERLKHGTFGPVHFRNQ	P50
CA07/VIC210	PB2	TFGPVHFRNQVKIRRRVD	P50
CA07/VIC210	PB2	DLSAKEAQDVIMEVVFPN	P50
CA07/VIC210	PB2	DVIMEVVFPNEVGARILT	P50
CA07/VIC210	PB2	LMVAYMLERELVRKTRFL	P50
CA07/VIC210	PB2	SIDRFLRVRDQRGNVLLS	P50
CA07/VIC210	PB2	RDQRGNVLLSPEEVSETQ	P50
CA07/VIC210	PB2	ITYSSSMMWEINGPESVL	P50
CA07/VIC210	PB2	WEINGPESVLVNTYQWII	P50
CA07/VIC210	PB2	SGFVRTLFQQMRDVLGTF	P50
CA07/VIC210	PB2	GPALSINELSNLAKGEKA	P50
CA07/VIC210	PB2	LSNLAKGEKANVLIGQGD	P50
CA07/VIC210	PB2	KANVLIGQGDVVLVMKRK	P50
CA07/VIC210	PB2	GDVVLVMKRKRDSSILTD	P50
CA07/VIC210	PB2	RKRDSSILTDSQTATKRI	P50
CA07/VIC210	PB2	SILTDSQTATKRIRMAIN	P50

Figure. S3. Influenza peptide pools. Peptides were derived from the proteome of the H1N1pdm09 strain, A/California/07/2009 (CA), or the seasonal A/Perth/16/2009-like H3N2 strain, A/Victoria/210/2009 (Vic). Peptide sequences were composed of 18 amino acids with a 10 amino acid overlap with each subsequent peptide, spanning the entire length of each viral protein. Individual peptides from each viral strain were combined into peptide pools for each protein based on the region of the protein from which the peptide was derived. Homologous peptide sequences between the two strains were combined into their own peptide pools for each viral protein. Regions of each protein which compose each of the 50 peptide pools (P) are depicted. Blue, red, and purple regions represent unique sequences for each viral strain. Tan and green regions represent portions of each protein in which both viruses share 100% homology. The regions depicted in a horizontal line represent portions of the A/Victoria/210/2009 proteome for which no amino acid sequence was available.

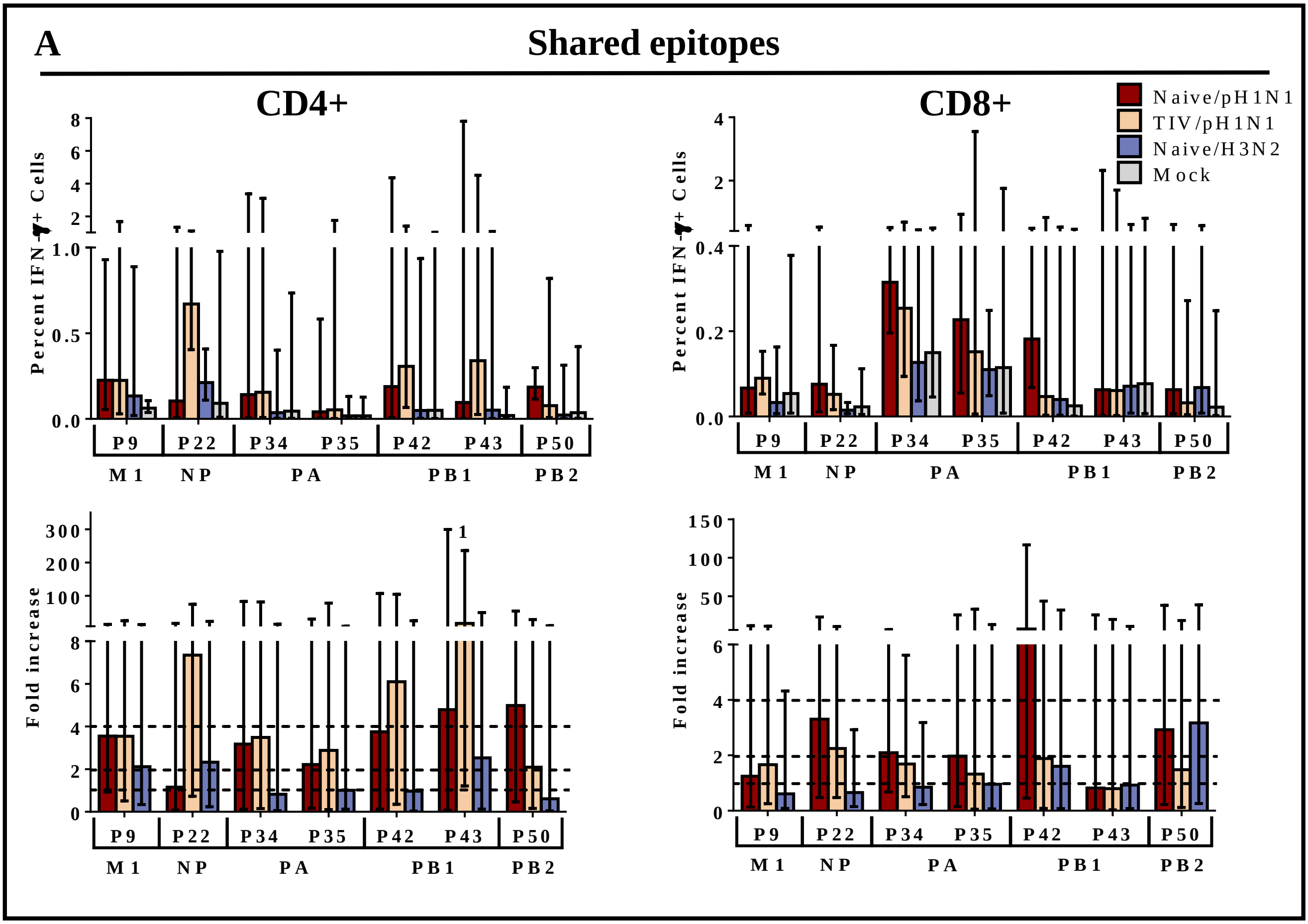

A
Shared epitopes
CD4+
CD8+
Naive/pH1N1
TIV/pH1N1
Naive/H3N2
Mock
Percent IFN-γ+ Cells
Fold increase
P9
P22
P34
P35
P42
P43
P50
M1
NP
PA
PB1
PB2

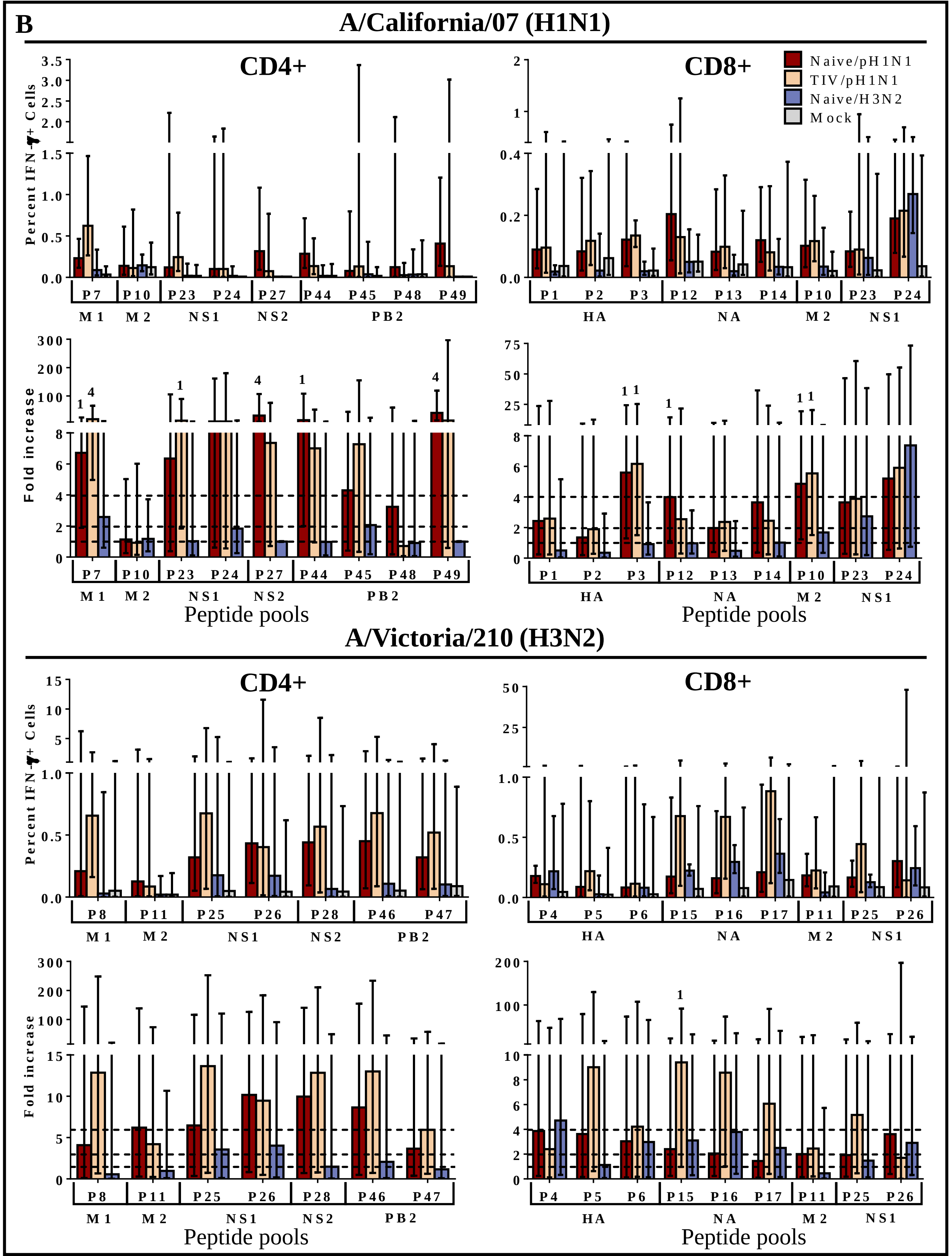

B
A/California/07 (H1N1)
CD4+
CD8+
Naive/pH1N1
TIV/pH1N1
Naive/H3N2
Mock
Percent IFN-γ+ Cells
Fold increase
P7 P10 P23 P24 P27 P44 P45 P48 P49
M1 M2 NS1 NS2 PB2
P1 P2 P3 P12 P13 P14 P10 P23 P24
HA NA M2 NS1
Peptide pools
A/Victoria/210 (H3N2)
CD4+
CD8+
P8 P11 P25 P26 P28 P46 P47
M1 M2 NS1 NS2 PB2
P4 P5 P6 P15 P16 P17 P11 P25 P26
HA NA M2 NS1
Peptide pools

Figure. S4. T cell responses to additional influenza peptide pools. Splenocytes were collected from naïve ferrets infected intranasally with A/NY/21/2009 (Naïve/H1N1pdm09, n=4), A/Perth/16/2009 (Naïve/H3N2, n=4), or mock infected ferrets (Naïve/Mock, n=4) 14d post infection. Splenocytes were also collected from ferrets vaccinated with commercial 2011-2012 TIV, challenged with A/NY/21/2009 (TIV/H1N1pdm09, n=4). Splenocytes were stimulated with peptide pools (p) derived from regions of each influenza protein from A/California/07/2009 (H1N1pdm09; A) or the A/Perth/16/2009-like seasonal H3N2 strain, A/Victoria/210/2009 (B). T cells producing IFN-γ in response to peptide stimulation were assessed by flow cytometry. The percent IFN-γ+ cells and fold-increase over mock infected animals are depicted. Responses to peptide sequences sharing 100% homology between both viruses were determined (A). Peptide pools to which lower percentages of CD4+ and CD8+ T cells responded are also represented.
Responses significantly [1]greater ($p < 0.05$) than Mock infected ferrets were considered modest responses, [2]2-fold greater were considered moderate, and [4]4-fold greater were considered substantial. Dotted lines indicate 1-, 2-, and 4-fold increases over mock infected controls. Error bars represent SEM.

CD4 IFN-γ+ Cells – All peptides

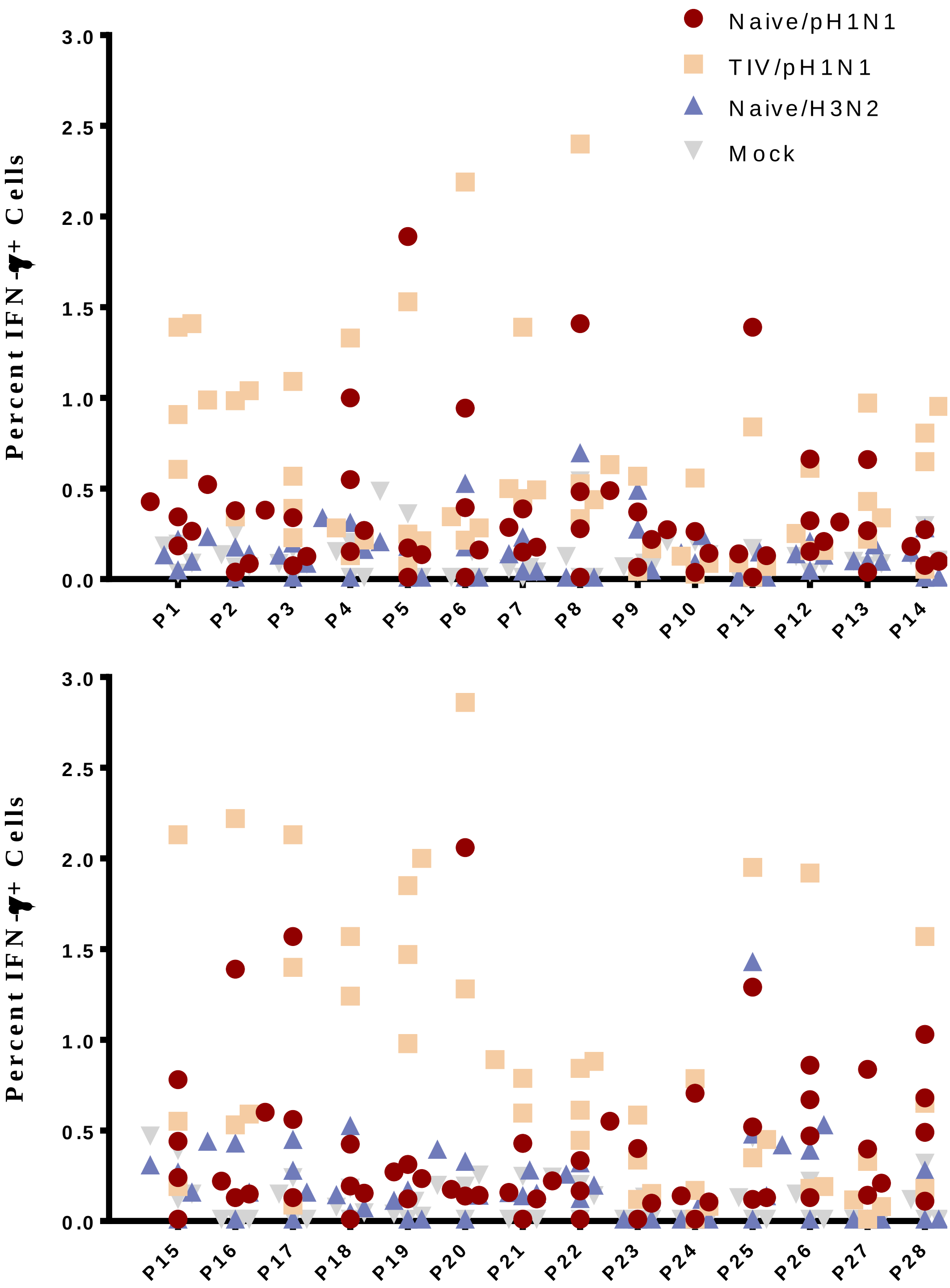

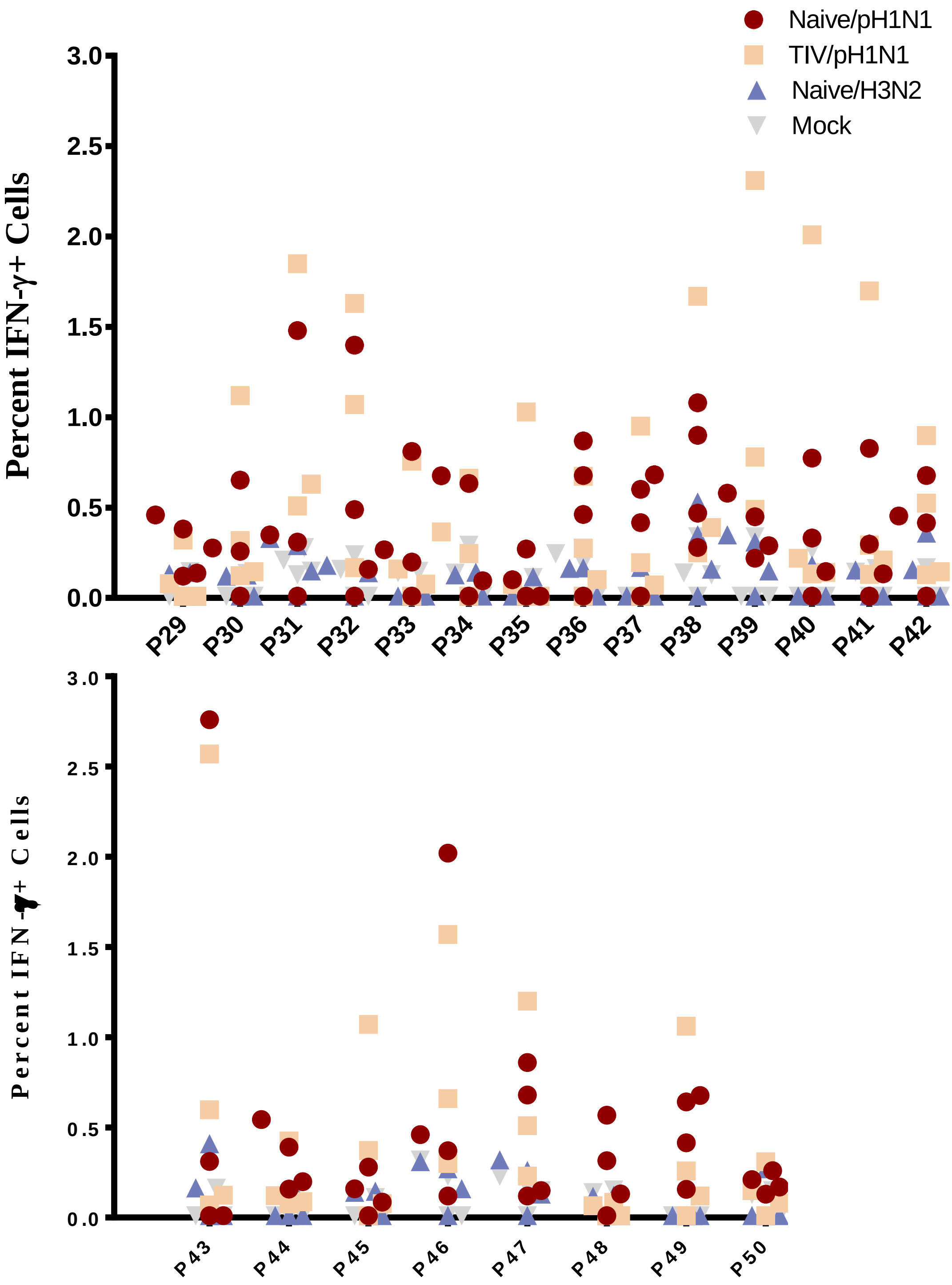

Naive/pH1N1
TIV/pH1N1
Naive/H3N2
Mock
Percent IFN-γ+ Cells
3.0
2.5
2.0
1.5
1.0
0.5
0.0
P29
P30
P31
P32
P33
P34
P35
P36
P37
P38
P39
P40
P41
P42
Percent IFN-γ+ Cells
P43
P44
P45
P46
P47
P48
P49
P50

Figure. S5. Individual ferret CD4+ T cell responses to influenza peptide pools. Splenocytes collected were collected from naïve ferrets infected with A/NY/21/2009 (Naïve/H1N1pdm09), A/Perth/16/2009 (Naïve/H3N2), or mock infected (Naïve/Mock) 14d post-infection. Splenocytes were also collected from ferrets vaccinated with commercial 2011-2012 TIV, challenged with A/NY/21/2009 (TIV/H1N1pdm09). Splenocytes were stimulated with peptide pools (p) derived from influenza proteins of A/California/07/2009 and A/Victoria/210/2009. The frequency of CD4+ T cells producing IFN-γ in response to peptide stimulation were assessed by flow cytometry.

CD8 IFN-γ+ Cells – All peptides

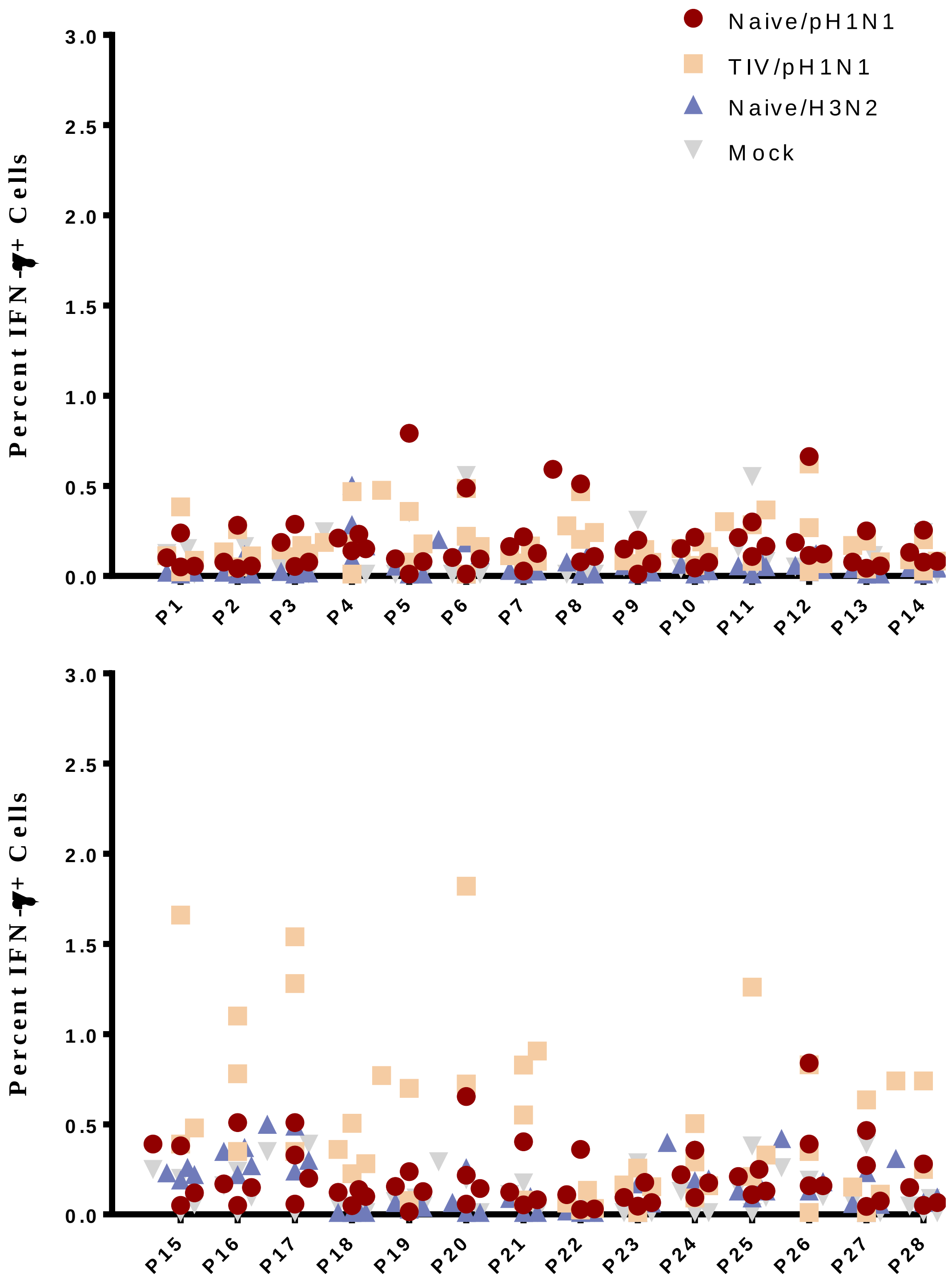

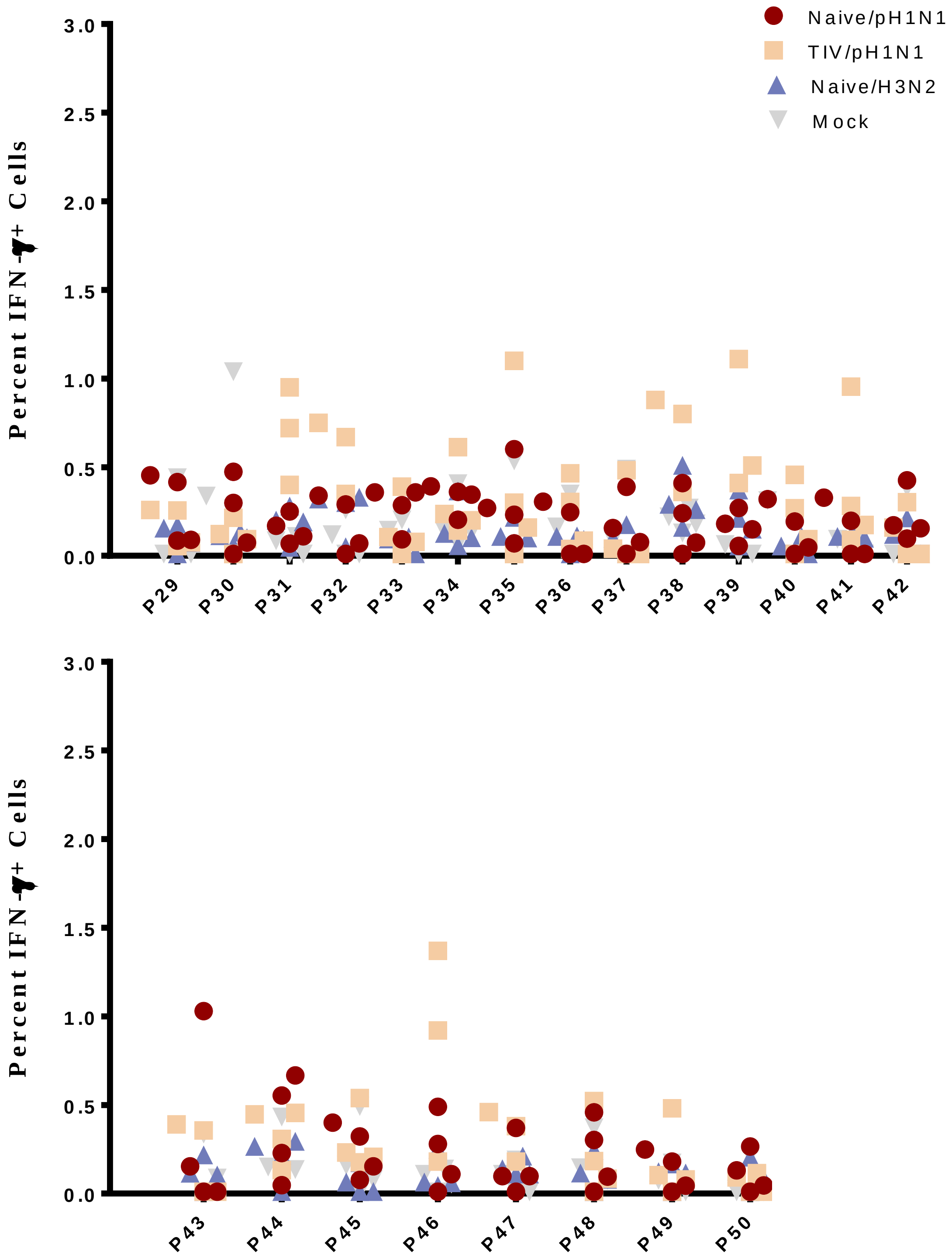
Naive/pH1N1
TIV/pH1N1
Naive/H3N2
Mock
Percent IFN-γ+ Cells
3.0
2.5
2.0
1.5
1.0
0.5
0.0
P29
P30
P31
P32
P33
P34
P35
P36
P37
P38
P39
P40
P41
P42
Percent IFN-γ+ Cells
3.0
2.5
2.0
1.5
1.0
0.5
0.0
P43
P44
P45
P46
P47
P48
P49
P50

Figure. S6. Individual ferret CD8+ T cell responses to influenza peptide pools. Splenocytes collected were collected from naïve ferrets infected with A/NY/21/2009 (Naïve/H1N1pdm09), A/Perth/16/2009 (Naïve/H3N2), or mock infected (Naïve/Mock) 14d post-infection. Splenocytes were also collected from ferrets vaccinated with commercial 2011-2012 TIV, challenged with A/NY/21/2009 (TIV/H1N1pdm09). Splenocytes were stimulated with peptide pools (p) derived from influenza proteins of A/California/07/2009 and A/Victoria/210/2009. The frequency of CD8+ T cells producing IFN-γ in response to peptide stimulation were assessed by flow cytometry.

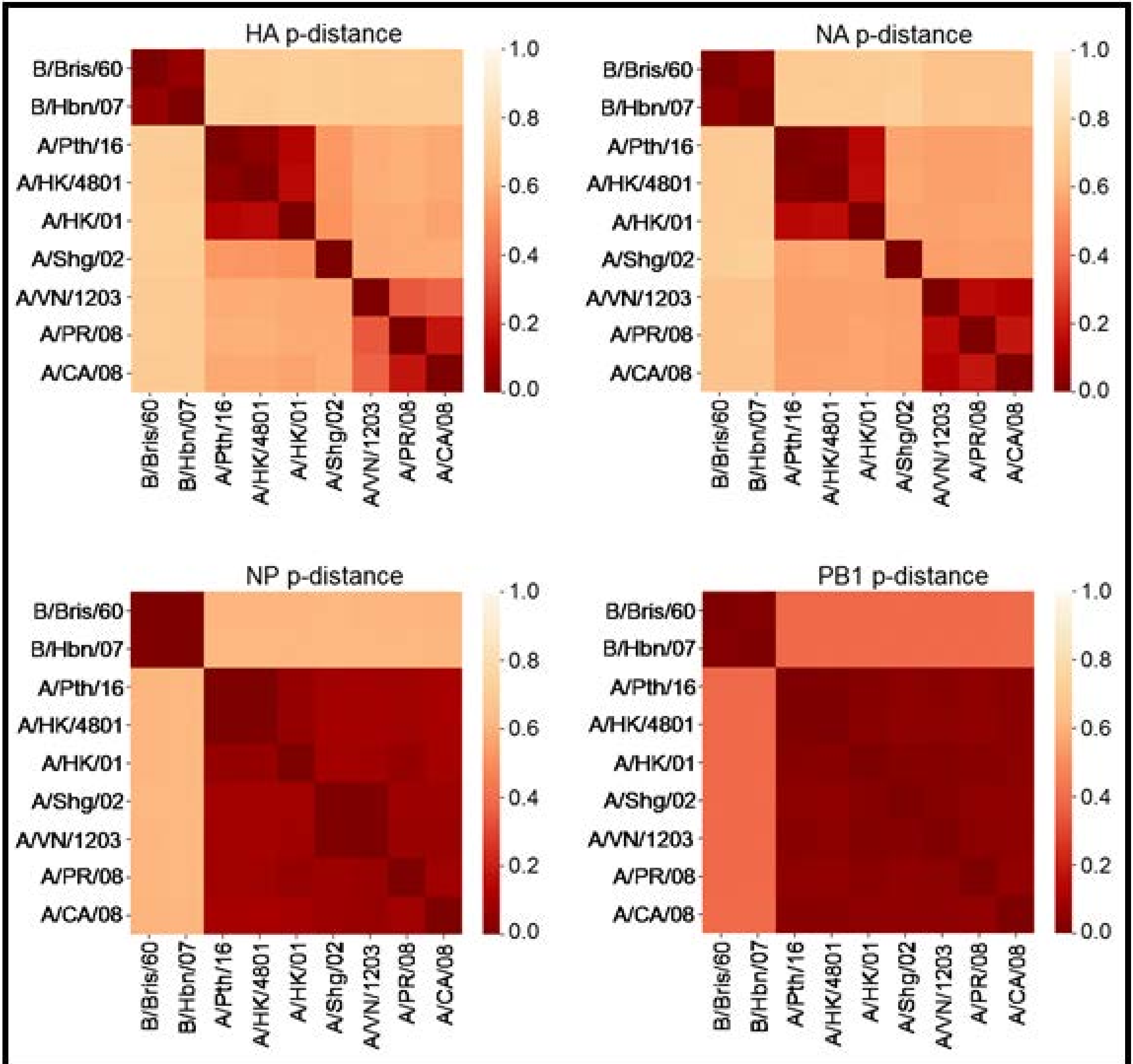
HA p-distance
NA p-distance
NP p-distance
PB1 p-distance
B/Bris/60
B/Hbn/07
A/Pth/16
A/HK/4801
A/HK/01
A/Shg/02
A/VN/1203
A/PR/08
A/CA/08
1.0
0.8
0.6
0.4
0.2
0.0

Figure. S7. Assessment of protein homology of viral proteins dominating the influenza response. Amino acid homology of the viral proteins which tended to dominate the responses to the influenza peptide pools were analyzed against a selection of contemporary and historical influenza strains, constructing p-distance heat maps to determine protein conservation.
